# *Porphyromonas gingivalis*-odontogenic infection is the potential risk for progression of nonalcoholic steatohepatitis-related neoplastic nodule formation

**DOI:** 10.1038/s41598-023-36553-y

**Published:** 2023-06-08

**Authors:** Shinnichi Sakamoto, Atsuhiro Nagasaki, Madhu Shrestha, Tomoaki Shintani, Atsushi Watanabe, Hisako Furusho, Kazuaki Chayama, Takashi Takata, Mutsumi Miyauchi

**Affiliations:** 1 Department of Oral and Maxillofacial Pathobiology, Graduate School of Biomedical and Health Sciences, Hiroshima University, 1-2-3 Kasumi, Minami-Ku, Hiroshima, Hiroshima 734-8551 Japan; 2 Division of Pathology, Department of Diagnostic and Therapeutic Sciences, Meikai University School of Dentistry, Sakado, Japan; 3 Division of Molecular and Regenerative Prosthodontics, Tohoku University Graduate School of Dentistry, Sendai, Japan; 4 Department of Diagnostic Sciences, Texas A&M University School of Dentistry, Dallas, TX USA; 5 Center of Oral Clinical Examination, Hiroshima University Hospital, Hiroshima, Japan; 6 Laboratory of Research Advancement, National Center for Geriatrics and Gerontology, Research Institute, Obu, Japan; 7 Collaborative Research Laboratory of Medical Innovation, Hiroshima University, Hiroshima, Japan; 8 Research Center for Hepatology and Gastroenterology, Hiroshima University, Hiroshima, Japan; 9 RIKEN Center for Integrative Medical Sciences, Yokohama, Japan; 10 Shunan University, 843-4-2 Gakuendai, Shunan, Japan

**Keywords:** Cancer, Diseases, Pathogenesis

## Abstract

*Porphyromonas gingivalis* (*P.g.*), a major periodontal pathogen is a known risk factor for various systemic diseases. However, the relationship between *P.g.* and nonalcoholic steatohepatitis (NASH)-related hepatocellular carcinoma (HCC) is unclear. Thus, we aimed to elucidate whether *P.g.*-odontogenic infection promotes NASH-related HCC development/progression and to clarify its mechanism. Using high-fat diet (HFD)-induced NASH mouse model, *P.g.* was infected odontogenically. After 60 weeks of infection, tumor profiles were examined. Chow diet (CD) groups were also prepared at 60 weeks. Nodule formation was only seen in HFD-mice. *P.g.*-odontogenic infection significantly increased the mean nodule area (P = 0.0188) and tended to promote histological progression score after 60 weeks (P = 0.0956). Interestingly, *P.g.* was detected in the liver. HFD-*P.g.* (+) showed numerous TNF-α positive hepatic crown-like structures and 8-OHdG expression in the non-neoplastic liver. In *P.g.*-infected hepatocytes, phosphorylation of integrin β1 signaling molecules (FAK/ERK/AKT) was upregulated in vitro. In fact, total AKT in the liver of HFD-*P.g.* (+) was higher than that of HFD-*P.g.* (−). *P.g.*-infected hepatocytes showed increased cell proliferation and migration, and decreased doxorubicin-mediated apoptosis. Integrin β1 knockdown inhibited these phenotypic changes. *P.g.*-odontogenic infection may promote the progression of neoplastic nodule formation in an HFD-induced NASH mouse model via integrin signaling and TNF-α induced oxidative DNA damage.

## Introduction

Nonalcoholic steatohepatitis (NASH) is a metabolic syndrome phenotype in the liver with lobular inflammation and hepatocellular ballooning besides fat deposition, and patients account for 3–5% of cases worldwide^1^. It is a progressive disease that may lead to liver cirrhosis and NASH-related hepatocellular carcinoma (HCC), thus making it an emerging critical health problem^1^.

HCC is the second leading cause of cancer-related death^2^. It has been reported that in the comparison of a total of 158,347 liver transplant candidate profiles from 2002 to 2016, the proportion of HCC patients among liver transplantation candidates increased from 6.4% in 2002 to 23% in 2016. NASH was the rapidly increasing cause of HCC in liver transplant candidates, with an 11.8-fold increase, whereas hepatitis C and alcoholic liver disease remained stable, and hepatitis B decreased by 3.1 times^3^. Furthermore, a UK team reported that nonalcoholic fatty liver diseases showing a progressive spectrum to simple fatty liver, NASH, and cirrhosis have become the most common cause of HCC^4^. In the future, NASH may become the leading cause of HCC given the rising rates of obesity; thus, NASH is an important disease that requires prevention and early diagnosis/therapeutic intervention worldwide.

Recent studies have shown that intestinal bacterial lipopolysaccharide (LPS) translocated from the portal vein due to dysbiosis accompanying obesity was related to NASH pathogenesis^2,5,6^. In addition, intestinal bacteria and LPS cause HCC development via Toll-like receptor 4 activation^7^.

*Porphyromonas gingivalis* (*P.g.*), a major periodontal pathogen, is a risk factor for systemic diseases including preterm birth, Alzheimer’s disease and NASH^8–11^. *P.g.* can be detected in various organs such as the liver and esophagus^9,10,12^. We previously reported that *P.g.*-odontogenic infection promoted inflammation and fibrosis in the liver of a high-fat diet (HFD)-induced NASH mouse model^9^. We also clarified that *P.g.*-odontogenic infection promoted liver fibrosis via transforming growth factor-β1 and galectin-3 production^13^. Moreover, *P.g.*-induced liver fibrosis and inflammation in NASH was inhibited by elimination of *P.g.*-odontogenic infection^10^. However, as far as we are aware, no reports have mentioned the relationship between *P.g.*-odontogenic infection and NASH-related HCC. Therefore, this study aimed to elucidate whether *P.g.*-odontogenic infection promotes NASH-related HCC development/progression and to explain its mechanism.

## Results

### Tumor profiles in HFD-induced NASH mouse model due to *P.g.*-odontogenic infection

At the 60 weeks after *P.g.*-odontogenic infection, the body and liver weight of the HFD-60w-*P.g.* (+) and HFD-60w-*P.g.* (−) groups were significantly increased compared with those of the CD-60w-groups (Supplementary Fig. 1a,b). In CD and HFD groups, there was no change in body and liver weight between mice with and without *P.g.*-odontogenic infection (Supplementary Fig. 1a,b).

**Figure 1:**
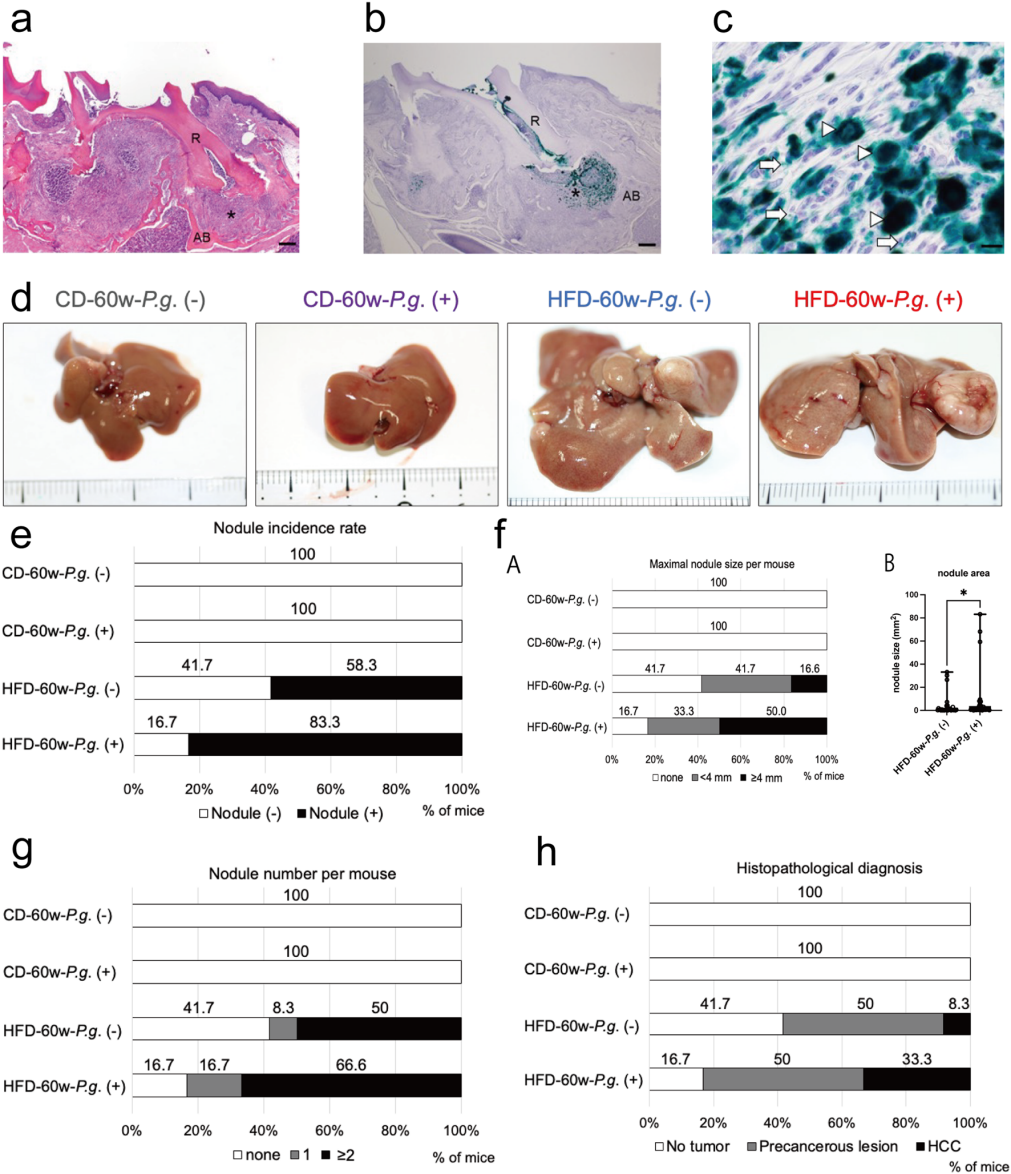
Effects of *P.g.*-odontogenic infection on NASH-related nodule formation. (**a**) Periapical granuloma (*) is formed in the root (R) apex areas of infected teeth. Alveolar bone (AB) (H&E staining); magnification, × 40; scale bar, 200 µm. (**b**) IHC of *P.g.*; *P.g.*-positive granules accumulate in pulpal tissue and periapical granuloma (*). Root (R), Alveolar bone (AB); magnification, × 40; scale bar, 200 µm. (**c**) *P.g.* are phagocytosed in macrophages (arrow heads) or separately distributed in intercellular spaces (arrows); magnification, × 1000; scale bar, 10 µm. (**d**) Representative macroscopic feature of the liver in each group. (**e**) Nodule incidence rate in each group; open bar, rate of mice without nodules; black bar, rate of mice with nodules. (**f**) (**A**) The diameter (mm) of the maximal nodule in each mouse is categorized as no tumor (open bar), < 4 mm (gray), and ≥ 4 mm (black). (**B**) The area (mm^2^) of each nodule in HFD-60W*-P.g.* (−) and *P.g*. (+) was calculated and compared among them (*P < 0.05: Mann–Whitney test). (**g**) The number of nodules in each mouse is categorized (no tumor, open bar; 1, gray bar; and ≥ 2, black bar) and then compared. (**h**) Histopathological analysis of nodules; open bar, no tumor; gray bar, precancerous lesions; black bar, HCC. (**e–h**) CD-*P.g.* (−) group, n = 9; CD-*P.g.* (+) group, n = 9; HFD-*P.g.* (−) group, n = 12; HFD-*P.g.* (+) group, n = 12). *NASH* nonalcoholic steatohepatitis, *HCC* hepatocellular carcinoma; *CD-P.g.* chow diet *Porphyromonas gingivalis* group, *HFD-P.g.* high-fat diet *Porphyromonas gingivalis* group.

The alveolar bones of the first molar root apices of all CD/HFD-*P.g*. (+) groups were markedly destroyed, and periapical granulomas were formed (Fig. 1a). No significant difference was found in the condition of periapical granulomas between the CD-*P.g*. (+) and HFD-*P.g.* (+) groups. Immunohistochemically, numerous *P.g.-*positive granules were found in the infected pulp and periapical granulomas (Fig. 1b). Figure 1c shows a high-power view of the periapical granuloma. Numerous *P.g.* were phagocytosed by Mφ (arrowheads), but some *P.g.* (arrows) were diffusely distributed among the cells.

Figure 1d shows the macroscopic findings of the liver. In both HFD-60w-*P.g.* (+) and (−) groups, the livers were large and yellowish because of fatty deposition. Meanwhile, the CD-60w groups generally had small and reddish-brown livers.

The HFD-60w-*P.g.* (+) group had larger liver nodules than the HFD-60w-*P.g.* (−) group. Macroscopically, nodule incidence rate tended to be higher in the HFD-60w-*P.g.* (+) group than that of the HFD-60w-*P.g.* (−) group, 83.3% (10/12) and 58.3% (7/12), respectively (Fig. 1e) (P = 0.3707, Fisher’s exact test between the HFD-60w-*P.g.* (−) group and the *P.g.* (+) group). However, no mice in the CD-60w-*P.g.* (−) and CD-60w-*P.g.* (+) groups had nodules. This indicates that *P.g.*-odontogenic infection may contribute to promoting the neoplastic nodule formation only in HFD-induced NASH livers (Fig. 1e). The maximal nodule size was categorized as no tumor, < 4 mm or ≥ 4 mm because all of HCC were ≥ 4 mm. Since the maximal nodule size in the HFD*-*60w-*P.g.* (+) group tended to be larger (50% of them were ≥ 4 mm) than that in HFD*-*60w-*P.g.* (−) (16.6%) (Fig. 1fA), the mean areas of all nodules were compared between the HFD-60w-*P.g.* (−) group and HFD-60w-*P.g.* (+) group. The mean nodule area of HFD-60w-*P.g.* (+) group was significantly larger than that of the HFD-60w-*P.g.* (−) group (P = 0.0188; Fig. 1fB). Because 66.6% of the HFD-60w-*P.g.* (+) group (8/12 mice) had ≥ 2 nodules (Fig. 1g), the number of nodules formed in individual mouse was compared between the HFD-60w-*P.g.* (−) group and HFD-60w-*P.g.* (+) group. The mean number of nodules in HFD-60w-*P.g.* (+) group tended to be increased than that of the HFD-60w-*P.g.* (−) group (P = 0.3187; Supplementary Fig. 2a). Almost no nodule formation was observed at 48 weeks (0/5 in HFD-48w-*P.g.* (−) and 1/10 in HFD-48w-*P.g.* (+)) (data not shown).

Histologically, liver carcinogenesis is a multistep process^14^. In mouse HCC, it sequentially develops from precancerous lesions, including the focus of cellular alteration (early stage of hepatocarcinogenesis) and hepatocellular adenoma (late stage) (Supplementary Fig. 2b)^15^.

Precancerous lesions and HCC were only seen in HFD groups. Most of the nodules in the HFD-60w-*P.g.* (−) group (50%, 6/12 mice) were precancerous lesions, but HCC was found in only in 8.3% (1/12 mice). By contrast, precancerous lesion in the HFD*-*60w*-P.g.* (+) group was 50% (6/12 mice), being similar to that in the HFD-60w-*P.g.* (−) group. However, interestingly 33.3% of HFD*-*60w*-P.g.* (+) group (4/12 mice) had HCC (Fig. 1h). Histopathological tumor progression procession score was also compared between the HFD-60w-*P.g.* (−) group and the HFD-60w-*P.g.* (+) group. *P.g.*-odontogenic infection showed tendency to promote histopathological progression of neoplastic nodule (P = 0.0956; Supplementary Fig. 2c).

Trabecular HCC is representative histology (Supplementary Fig. 2dA). In HFD-60w-*P.g.* (+) mice, a case of trabecular HCC with hepatoblastoma-like small cell area (Supplementary Fig. 2dB) was observed.

### Changes in NASH condition in the non-neoplastic liver area due to *P.g.*-odontogenic infection

**Figure 2:**
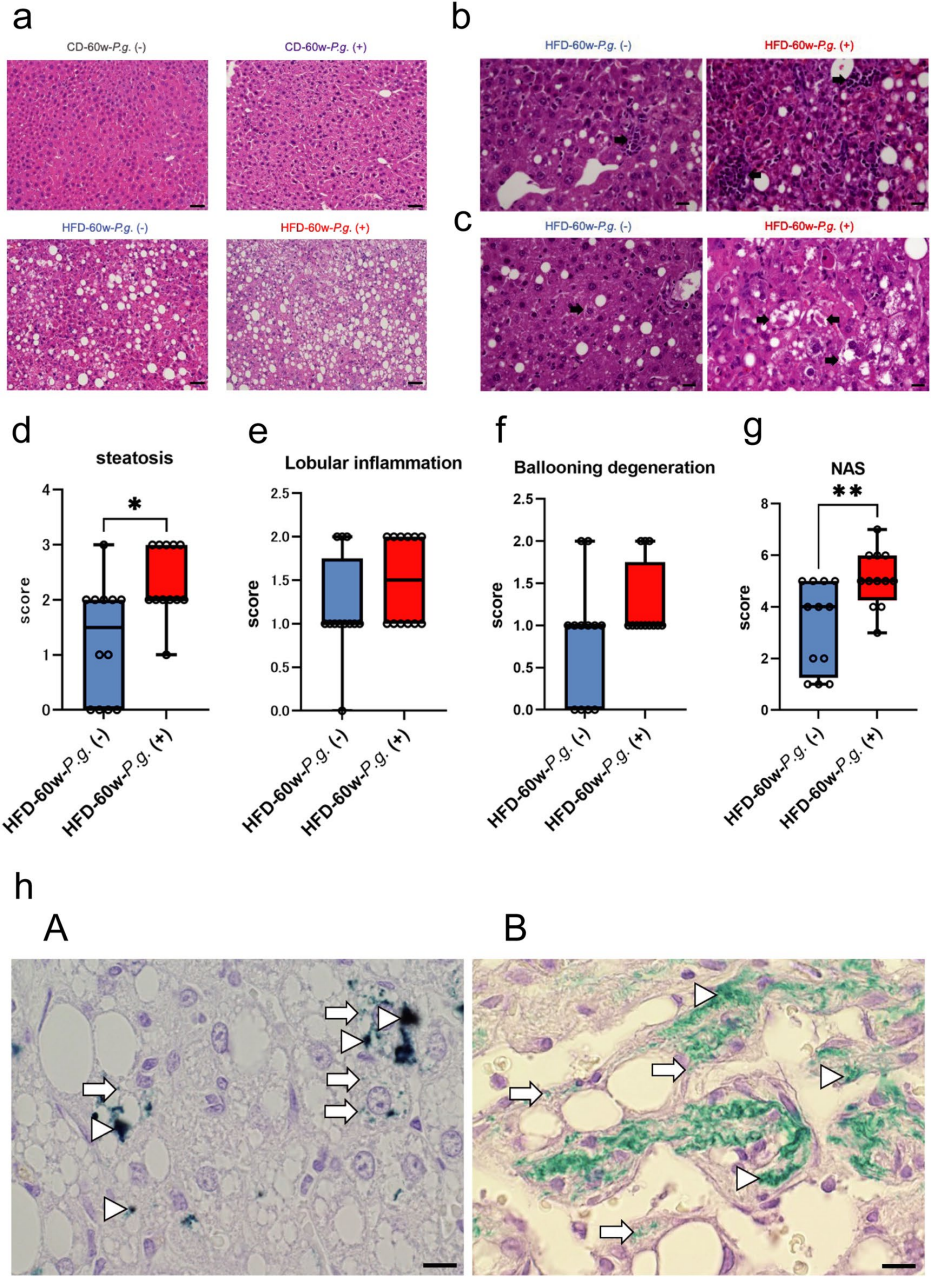
Histopathological changes in the non-neoplastic liver of the HFD-*P.g.* (+) group. (**a**) Fatty degeneration is more prominent in the HFD-60w-*P.g.* (+) group. Hematoxylin and eosin (H&E) staining; magnification, × 200; scale bar, 50 µm. (**b**) Increased number of lobular inflammation (arrows); H&E staining; magnification, × 200; scale bar, 50 µm. (**c**) Hepatocyte ballooning (arrows); magnification, × 400; scale bar, 20 µm. (**d**) Steatosis scores (0–3) (*P < 0.05, Mann–Whitney test). (**e**) Lobular inflammation scores (0–3). (**f**) Ballooning degeneration scores (0–2). (**g**) NAS was calculated and compared among experimental groups (**P < 0.01, Mann–Whitney test). (**d–g**) CD-60w-*P.g.* (−) group, n = 9; CD-60w-*P.g.* (+) group, n = 9, HFD-60w-*P.g.* (−) group; n = 12; and HFD-60w-*P.g.* (+) group, n = 12). (**h**) IHC of *P.g.* in (**A**) non-neoplastic liver and (**B**) HCC of the HFD-60w-*P.g.* (+) group. *P.g.* clusters (arrowheads) or scattered *P.g.* (arrows) are detected in both non-neoplastic liver and HCC. Magnification, × 1000; scale bar, 10 µm. *CD-P.g.* chow diet *Porphyromonas gingivalis* group, *HFD-P.g.* high-fat diet *Porphyromonas gingivalis* group, *HCC* hepatocellular carcinoma, *NAS* NAFLD Activity Score.

We investigated the histological changes underlying NASH condition of the liver in HFD groups. After 60 weeks of *P.g.*-odontogenic infection, fatty degeneration was remarkable in the HFD-60w-*P.g.* (+) group (Fig. 2a). Lobular inflammation (Fig. 2b) and hepatocyte ballooning (Fig. 2c) were also severe in the HFD-60w-*P.g.* (+) group. The NAFLD activity score (NAS), sum of the steatosis, lobular inflammation, and ballooning degeneration score in patients with NAFLD, was applied to quantitatively evaluate NASH activity in the animal liver. (Supplementary Table 1)^16^. The steatosis score was significantly higher in the HFD-60w-*P.g.* (+) group than that of HFD-60w-*P.g*. (−) group (P = 0.0111; Fig. 2d). The HFD-60w-*P.g.* (+) group also tended to show higher lobular inflammation (P = 0.2759; Fig. 2e) and ballooning degeneration scores (P = 0.1889; Fig. 2f) than those of HFD-60w-*P.g*. (−) group. The NAS of the HFD-60w-*P.g.* (+) group (5.1) was significantly higher than that of HFD-60w-*P.g.* (−) (3.3) group (P = 0.0068; Fig. 2g). Interestingly, *P.g.* and *P.g.*-clusters were immunohistochemically detected in non-neoplastic area of both the CD-60w-*P.g.* (+) and HFD-60w-*P.g.* (+) groups (Fig. 2hA). Furthermore, *P.g.* was also detected in HCC area (Fig. 2hB). These findings indicate that *P.g.*-odontogenic infection exacerbate pathological condition of the livers in HFD group, but not in normal livers of the CD group and suggest that *P.g.* is a potential key factor in progression of neoplastic nodule formation in the HFD-induced NASH mouse model.

### Changes in macrophages (Mφ), hepatic crown-like structures (hCLSs), and TNF-α expression in the non-neoplastic liver due to *P.g.*-odontogenic infection

hCLSs, which is composed of Mφ surrounding the degenerated hepatocytes (an indicator of NASH inflammation and fibrosis), is accepted to correlate not only the onset of chronic inflammation and fibrosis^17^ but also HCC development in NASH^18^. hCLSs can produce TNF-α, which plays a critical role in hepatocarcinogenesis^19^. Therefore, we considered that increased hCLSs with *P.g.*-infection might be associated with progression of nodule formation through TNF-α production.

**Figure 3:**
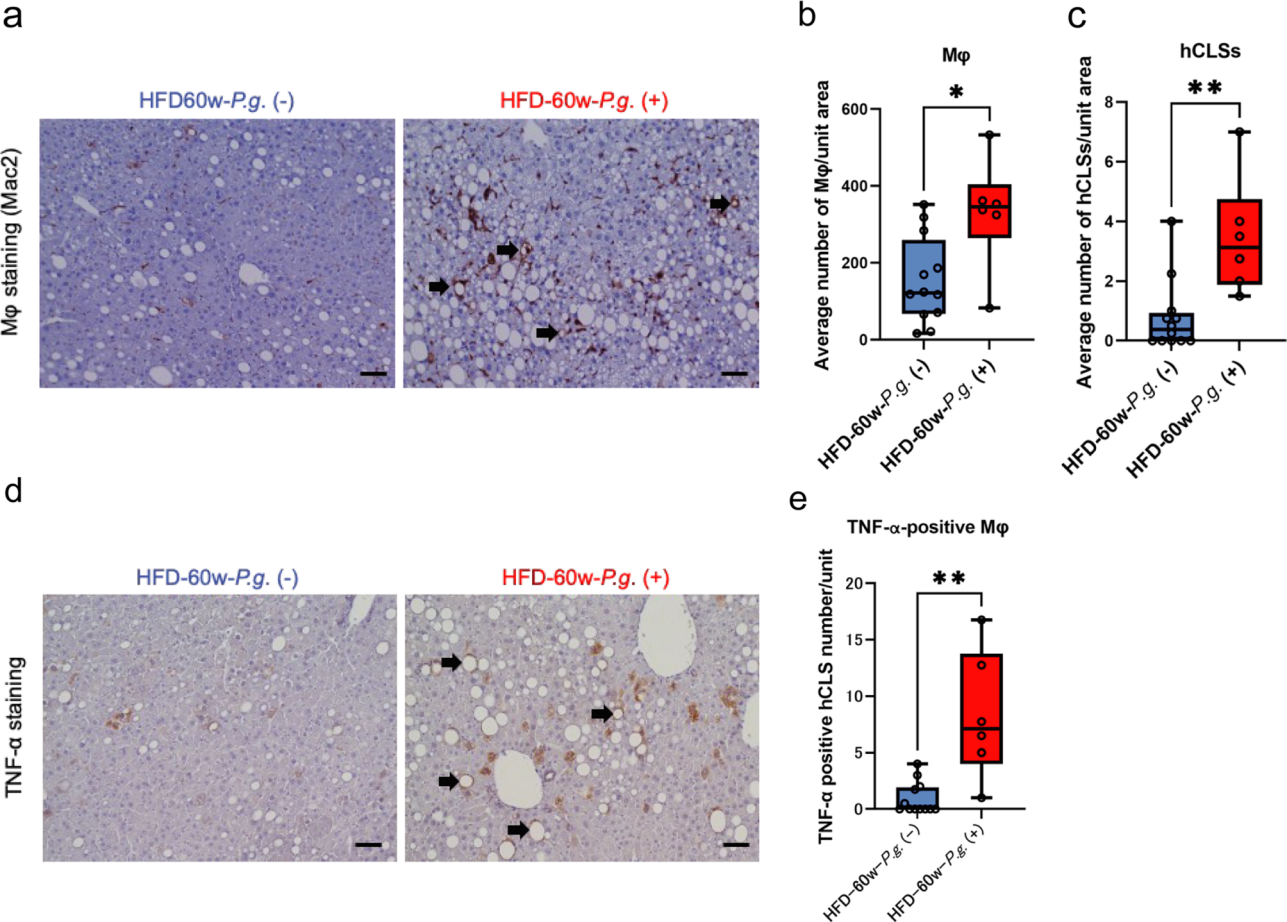
Effects of *P.g.*-odontogenic infection on the increase in the number of Mφ and hCLSs and TNF-α expression in the non-neoplastic liver. (**a**) IHC of Mac2 in the non-neoplastic liver tissue of HFD-60w-*P.g.* (−) and *P.g.* (+) group; magnification, × 200; scale bar, 50 µm. (**b**) Number of Mac2-poitive Mφ (*P < 0.05, Mann–Whitney test) and (**c**) Mac2-positive hCLSs (arrows) in non-neoplastic livers of HFD-60w-*P.g.* (−) and *P.g.* (+) group (**P < 0.01, Mann–Whitney test). (**d**) IHC for TNF-α in the non-neoplastic livers of HFD-60w-*P.g.* (−) and *P.g.* (+) group. Arrows represent TNF-α-positive hCLSs; magnification, × 200; scale bar, 50 µm. (**e**: Number of TNF-α-positive hCLSs in the non-neoplastic liver of HFD-60w-*P.g.* (−) and *P.g.* (+) group (**P < 0.01, Mann–Whitney test). (**a–e**) CD-60w-*P.g.* (−) group, n = 9; CD-60w-*P.g.* (+) group, n = 9; HFD-60w-*P.g.* (−) group, n = 12, and HFD-60w-*P.g.* (+) group, n = 6. *CD-P.g,* chow diet *Porphyromonas gingivalis* group, *HFD-P.g* high-fat diet *Porphyromonas gingivalis* group, *hCLS* hepatic crown-like structures, *TNF* tumor necrosis factor.

Immunohistochemically, the number of Mac2-positive Mφ in the non-neoplastic liver of the HFD-60w-*P.g.* (+) group was significantly higher than that of HFD-60w-*P.g*. (−) group (Fig. 3a,b; P = 0.0135). Furthermore, the number of hCLSs in the HFD-60w-*P.g.* (+) group was also significantly higher than that of HFD-60w-*P.g*. (−) group (Fig. 3a,c; P = 0.0036).

Figure 3d showed Immunohistochemistry of TNF-α in non-neoplastic liver. In the HFD-60w-*P.g.* (+) group, the hCLSs showing intensive TNF-α positivity were more abundantly scattered than in the HFD-60w-*P.g.* (−) group. Hepatocytes were weakly positive for TNF-α. The number of TNF-α-positive hCLSs in the HFD-60w-*P.g.* (+) group was significantly higher than that in HFD-60w-*P.g*. (−) group (P = 0.001; Fig. 3e). Moreover, IL-6 was checked because it is also known tumor promoting cytokines for NASH-related HCC^19^. IL-6 was significantly upregulated in HFD-60w-*P.g.* (+) group (P = 0.0408; Supplementary Fig. 3a,b). In our study, *P.g.-*induced exacerbation of NASH was significantly severe. NASH progression is said to be closely related to oxidative stress^20^. Therefore, we considered that oxidative stress caused by hCLSs-induced TNF-α may produce 8-OHdG to cause nodule formation.

### Changes in the oxidative DNA damage in the non-neoplastic liver due to *P.g.*-odontogenic infection

**Figure 4:**
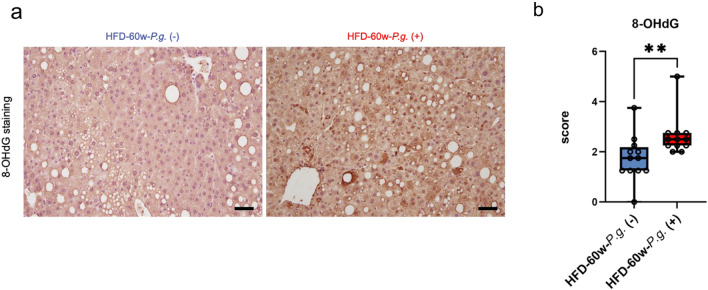
Effects of *P.g.*-odontogenic infection on the 8-OHdG expression in the non-neoplastic liver. (**a**) IHC of 8-OHdG in the non-neoplastic liver tissue of HFD-60w-*P.g.* (−) and *P.g.* (+) group; magnification, × 200; scale bar, 50 µm. (**b**) Score of 8-OHdG (*P < 0.05, **P < 0.01, Mann–Whitney test).

TNF-α is capable of causing oxidative DNA damage^21^. It has been reported that oxidative DNA damage triggered by various factors such as inflammation promotes hepatocarcinogenesis, and 8-hydroxy-2-deoxyguanosine (8-OHdG), oxidative DNA damage marker which can cause G: C to T: A mutation, is useful for predicting NASH-related HCC development^20,22^. As expected, 8-OHdG was significantly increased in HFD-60w-*P.g.* group (P = 0.0029; Fig. 4a,b). It was stained with both nuclei and cytoplasm. These results indicate that hCLSs-induced TNF-α upregulation caused by *P.g.*-odontogenic infection promote neoplastic nodule formation through mutagenic 8-OHdG in an HFD-induced NASH mouse model.

### Changes in the integrin–FAK pathway in hepatocytes due to *P.g.*-infection

Immunohistochemically, *P.g.* was mainly detected in hepatocytes in non-neoplastic liver of *P.g.* (+) groups. Interestingly, *P.g.* can bind to integrin α5β1 in oral epithelial cells leading to integrin signaling activation^23^. Furthermore, Integrin β1 signaling is involved in HCC development/progression^24,25^ and drug resistance in HCC^25^. Therefore, in this study, we focused on integrin β1-FAK signaling in nodule formation.

**Figure 5:**
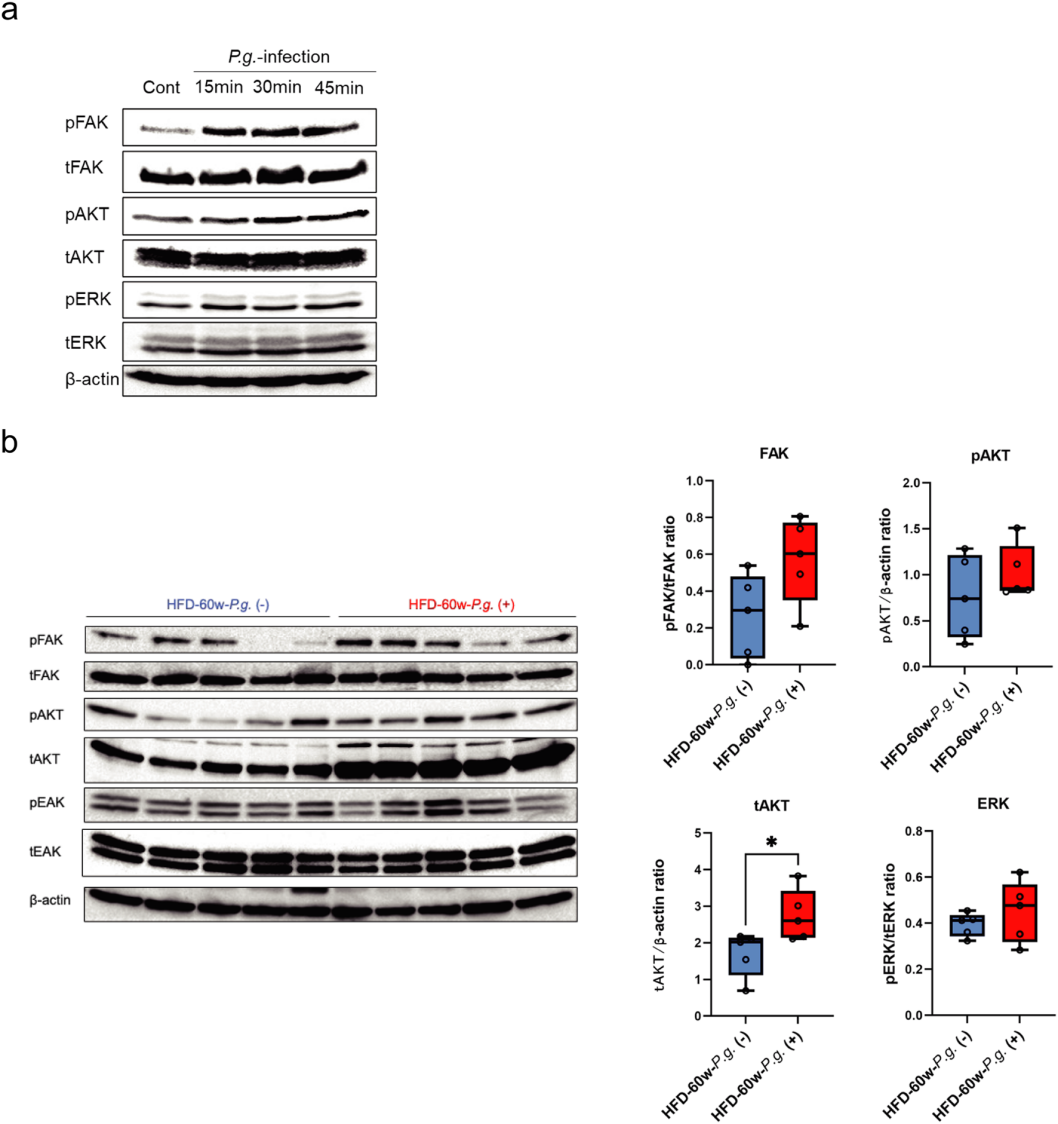
Effects of *P.g.*-infection on the activation of integrin β1-signaling molecule. (**a**) HC3716-hTERT cells were infected with *P.g.* at MOI of 100. After 15, 30, and 45 min of *P.g.*-infection, protein was collected, and phosphorylations of FAK, AKT, and ERK were confirmed by Western blotting. (**b**) Effects of *P.g.*-odontogenic infection on integrin β1-signaling molecule activation in non-neoplastic livers of HFD-60w-*P.g.* (+) group. FAK, AKT, and ERK phosphorylations were examined in the non-neoplastic livers of HFD-60w-*P.g.* (−) and *P.g.* (+) group (HFD-60w-*P.g.* (−) group, n = 5; HFD-60w-*P.g.* (+) group, n = 5) via Western blotting. The ratio of phosphorylated protein/total protein was calculated and compared using ImageJ software, ver.1.52a (https://imagej.net/ij/docs/index.html). Significant overexpression of total AKT is confirmed (*P < 0.05, Mann–Whitney test). β-actin is used as an internal control for phosphorylated AKT and total AKT. *CD-P.g* chow diet *Porphyromonas gingivalis* group, *HFD-P.g* high-fat diet *Porphyromonas gingivalis* group, *hCLSs* hepatic crown-like structures, *TNF* tumor necrosis factor.

Firstly, we analyzed the effects of *P.g.*-infection on FAK activation and its downstream AKT and ERK. The phosphorylations of FAK, AKT, and ERK were upregulated at 15, 30, and 45 min after infection (Fig. 5a). These molecules in non-neoplastic mouse liver tissue of HFD-60w-*P.g.* (−) and *P.g.* (+) group were also examined by Western blotting. Accordingly, these molecules tended to be activated in the HFD-60w-*P.g.* (+) group (pAKT P = 0.4206, pERK P = 0.5476, pFAK P = 0.0952; Fig. 5b). Interestingly, total AKT was significantly higher in the HFD-60w-*P.g.* (+) group than in the HFD-60w-*P.g.* (−) group (P = 0.0317; Fig. 5b).

### Changes in the phenotypes in hepatocytes through the integrin–FAK pathway activated by *P.g.*-infection

**Figure 6:**
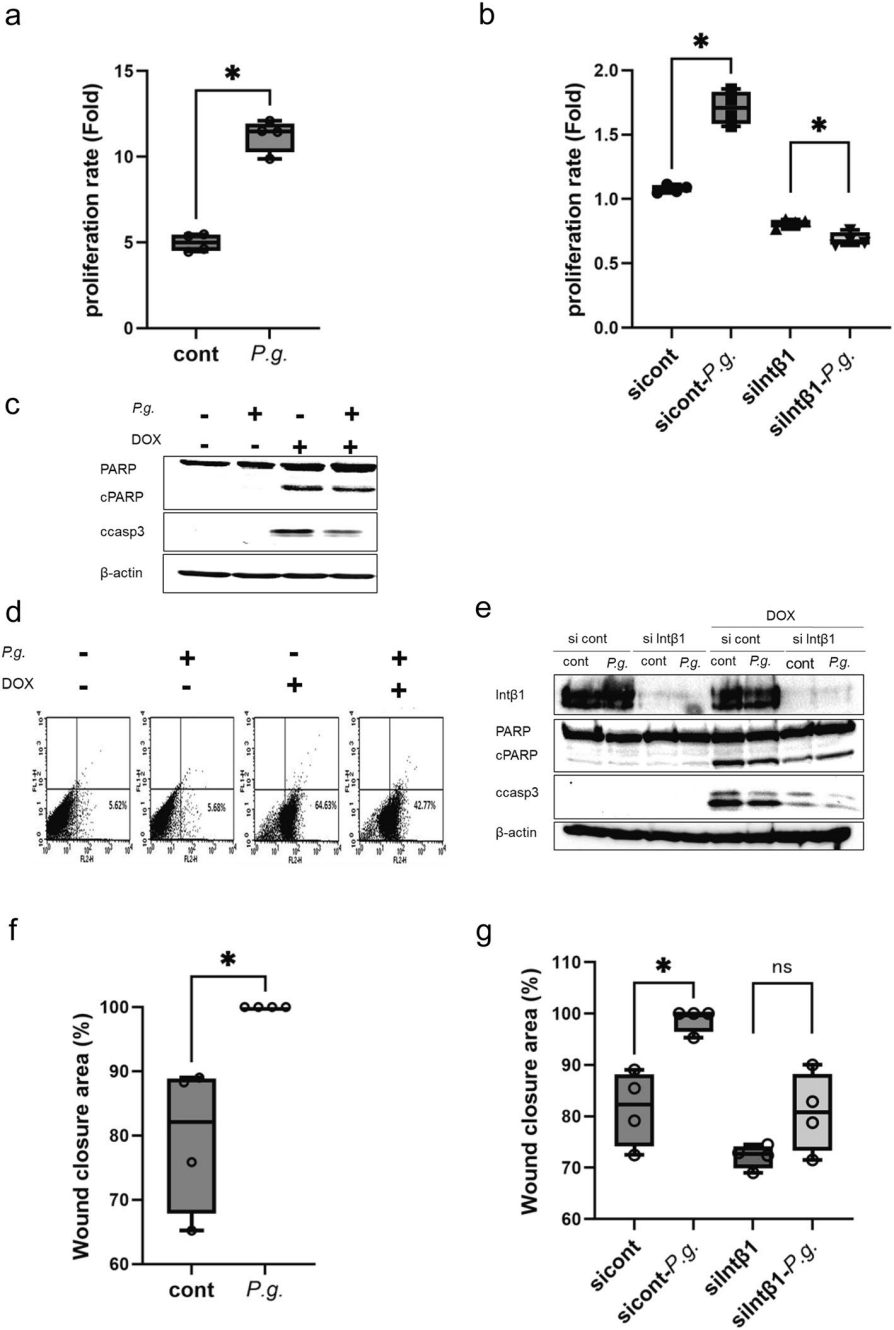
Effects of *P.g.*-infection on cell proliferation, anti-apoptosis, and migration. (**a**) HC3716-hTERT cells with *P.g.*-infection at MOI of 100 were seeded at 1 × 10^4^ cells/well in 24-well plates. The number of cells on days 1 and 5 of *P.g.*-infection was measured, and the growth rate was determined (*P < 0.05, Mann–Whitney test) (n = 4). (**b**) The effect of integrin β1 knockdown on cell proliferation rate was also evaluated (*P < 0.05, Mann–Whitney test) (n = 4). (**c**) One day after *P.g.*-infection of HC3716-hTERT at MOI of 100, Dox was applied for 18 h to determine the amount of cCASP3 and cPARP, which are markers of apoptosis, via Western blotting. (**d**) Flow cytometry was performed under the same conditions. (**e**) Similarly, cCASP3 and cPARP were detected via Western blotting with integrin β1 knockdown cells to confirm the importance of integrin signaling in the anti-apoptotic effect of *P.g.*-infection. (**f**) HC3716-hTERT cells were infected with *P.g.* at MOI of 100, and the closure rate at 24 h after scratch was compared with that in uninfected cells (*P < 0.05, Mann–Whitney test) (n = 4). (**g**) The effects of integrin β1 knockdown using siRNA were also evaluated (MOI of 100) (*P < 0.05, Mann–Whitney test) (n = 4). *CD-P.g* chow diet *Porphyromonas gingivalis* group, *HFD-P.g* high-fat diet *Porphyromonas gingivalis* group, *MOI* multiplicity of infection, *cCASP3* cleaved caspase, *cPARP* cleaved poly(ADP-ribose) polymerase cleavage.

Considering that AKT and ERK, downstream of FAK, are involved in cell proliferation, anti-apoptosis, and migration, the effects of *P.g.*-infection on hepatocyte proliferation, apoptosis, and migration were examined. *P.g.*-infected cells showed significant upregulation of cell proliferation (P = 0.0286; Fig. 6a). In integrin β1-knockdown cells, *P.g.*-induced cell proliferation was not seen, and cell proliferation was reversely downregulated (P = 0.0286; sicont vs sicont-*P.g.*, P = 0.0286; siIntβ1 vs siIntβ1-*P.g.*, Fig. 6b). Dox-induced cleavage of PARP and caspase-3 was attenuated by *P.g.*-infection, indicating the suppression of apoptosis (Fig. 6c). The proportion of Dox-induced apoptotic cells (64.63%) in non-infected cells decreased to 42.77% in *P.g.*-infected cells (Fig. 6d). In integrin β1-knockdown cells, no anti-apoptotic effect of *P.g.*-infection was observed (Fig. 6e). *P.g.*-infection caused a significant increase in migration capability via wound-healing assay (P = 0.0286; Fig. 6f). Meanwhile, in integrin β1-knockdown cells, *P.g.*-infection-induced significant upregulation of migration capability was not evident (P = 0.0286; sicont vs sicont-*P.g.*, P = 0.2; siIntβ1 vs siIntβ1-*P.g.*, Fig. 6g).

## Discussion

In this study, *P.g.*-odontogenic infection induced progression of neoplastic nodule formation in the liver of an HFD-induced NASH mouse model. To the best of our knowledge, this is the first study to confirm the role of *P.g.*-odontogenic infection in NASH-related neoplastic nodule formation.

In an HFD-induced NASH mouse model, NASH was induced only by HFD without the addition of chemicals or genetic modifications. As such, we consider that a pathological condition mimicking human NASH could be created. It has been reported that liver neoplastic nodule formation occurred in approximately half of C57B1/6J mice after 60 weeks of HFD feeding^26^. We also found neoplastic nodules in the liver of 66.7% (8/12 mice) of the mice in the HFD-60w-*P.g.* (−) group, indicating that it is appropriate as a research model to examine the effects of odontogenic infection of periodontal pathogens on NASH-related atypical/neoplastic nodule formation including HCC.

*P.g.* is a main periodontal pathogen that requires anaerobic conditions for growth. It is well accepted that *P.g.* is detected not only in deep periodontal pocket, but also in distant organ like arteriosclerosis plaque or brain suffering to Alzheimer disease, resulting in occurrence and/or exacerbation of systemic diseases. Moreover, *P.g.* was immunohistochemically detected in the tumor area of esophageal cancer and was associated with cancer differentiation, lymph node metastasis, and TNM stage^12^. It is reported that *P.g.* is detected in infected pulp chamber with periapical periodontitis as a second major bacterium next to *P. endodontalis* and associated with moderate to high cardiovascular risk^27^. Pereira et al. confirmed that *P.g.* is more prevalently detected together with *P. intermedia* in pulp chamber of endodontic-periodontal disease^28^. Therefore, in the present study, *P.g.* was selected as infecting bacterium, and to obtain anaerobic conditions suitable for *P.g.* growth, we applied *P.g.* from pulp chamber, which ideally provided the natural infection way of periapical periodontitis. Previously we confirmed that *P.g.* was alive inside the pulp chamber and reproduced itself over a long period to induce periapical periodontitis and exacerbated inflammation and fibrosis of NASH^9,10^. In the present study, after 60 weeks of *P.g.*-odontogenic infection, numerous *P.g.* were detected in the pulp and periapical granuloma and were distributed in the liver. The average NAS in the liver of HFD-60w-*P.g.* (+) group was significantly higher than that in HFD-60w-*P.g.* (−) group, indicating that periapical granuloma still functioned as reservoir of *P.g.* to aggravate NASH progression.

Interestingly, in CD mice with or without *P.g.-*odontogenic infection not only HCC but also tumor nodule formation indicating precancerous lesion was not observed.

Using the HFD-induced NASH mouse model with short period of *P.g.*-odontogenic infection (6 weeks), we previously confirmed that *P.g.*-odontogenic infection exacerbated inflammation/fibrosis of NASH, in which mechanism TLR2 upregulating and/or inflammasome activating by lipid accumulation in hepatocytes contributed to excessive production of proinflammatory cytokines.

In other words, fatty liver has an increased susceptibility to *P.g.*-LPS/lipoprotein.

Therefore, it is suggested that *P.g*.-odontogenic infection like periodontitis can more aggressively exacerbate NASH-related neoplastic nodule formation in obese individual than normal individuals. Actually, the mean nodule area of the HFD-60w-*P.g.* (+) was significantly larger than that of HFD-60w-*P.g.* (−) group (P = 0.0188), indicating *P.g.*-odontogenic infection accelerated neoplastic nodule formation including precancerous and HCC nodules. Unfortunately, we could not get statistical difference in HCC development rate. In fact, 4 out of 12 mice (33.3%) progressed to HCC in HFD-60w-*P.g.* (+) group while only one out of 12 mice (8.3%) had HCC in the HFD-60w-*P.g.* (−) group. Considering all of data together, it is indicated that *P.g.*-odontogenic infection is an important risk factor for neoplastic nodule formation in the NASH liver of HFD-induced NASH mouse model.

It is well accepted that proinflammatory cytokines like TNF-α also correlated with development and/or progression of inflammation related cancers such as HCC. We considered that proinflammatory cytokines produced in the NASH liver with *P.g.* infection is potential risk for nodule formation.

In this study, TNF-α positive hCLSs were significantly increased in the non-neoplastic liver in the HFD-60w-*P.g.* (+) group. TNF-α is associated with HCC, colitis-associated cancer, and gallbladder cancer. In the liver, TNF-α induces reactive oxygen species (ROS). ROS causes DNA damage, which initiates tumorigenesis^29^. Autocrine TNF-α was reported to act as a tumor promoter gene by promoting gallbladder cancer cell proliferation via the AKT/NF-κB/Bcl-2 pathway^30^. In mouse hepatocytes, TNF-α phosphorylated AKT, which plays an important role in the regulation of cell proliferation and apoptosis^31^. Moreover, ERK is another downstream molecule of TNF-α signaling pathway. ERK expression was attenuated in HCC progenitor cells isolated from DEN-treated TNFR1-knockout mice, in which HCC development was suppressed^32^. TNF-α is also known to promote FAK activation in epithelial cells, endothelial cells, fibroblasts and muscle cells to cause various cellular responses such as inflammatory cytokine, matrix metalloproteinase, or VCAM-1 production^33^. Especially, Corredor et al. have reported that TNF induces intestinal epithelial cells migration via TNFR2 initiated FAK phosphorylation^33,34^. Furthermore, Pokharel et al. mentioned that TNF-α promotes the production of 25-hydroxycholesterol (25HC), a lipid ligand for integrins, and induces pro-inflammatory response (CCL3 production) via integrin α5β1-FAK signaling^33^. Our in vitro experiments confirmed that *P.g.* directly activates integrin β1 signaling. Moreover, in vivo data also showed a tendency to activate FAK, AKT, and ERK. These data indicate that not only *P.g.* but also TNF-α may promote hepatocyte proliferation, anti-apoptosis and migration via integrin signaling. Nakagawa et al. hypothesized that NASH-HCC development was dependent on TNF-α produced by Mφ. The main source of TNF-α is hCLS, which is positively correlated with chronic inflammation and liver fibrosis^17^. In our study, IHC of TNF-α showed an increase in the number of TNF-α-positive hCLS in the HFD-60w-*P.g.* (+) group, indicating that TNF-α produced from Mφ and hCLS stimulated with *P.g.* may play an important role in progression of NASH-related nodule formation. TNF-α induces reactive oxygen species (ROS). ROS causes DNA damage which initiates tumorigenesis^29^. Maki et al. considered that DNA damage caused by HCV-induced chronic inflammation is associated with HCV-associated HCC development because 8-OHdG and CD68, Mφ marker, of noncancerous liver tissues of HCV-associated HCC patients was significantly upregulated compared to those of metastatic liver cancer patients, and significant positive correlations were confirmed between CD68-positive inflammatory cells and 8-OHdG in liver tissues of HCV-associated HCC patients^35^. Tanaka et al. performed immunostaining of 8-OHdG in cancerous and non-cancerous liver tissues of NASH-related HCC patients, and found that 8-OHdG was significantly increased compared to HBV-associated HCC and ALD-associated HCC patients suggesting that NASH-related HCC development is highly associated with oxidative DNA damage via ROS production^20^. It was also confirmed that 8-OHdG in NASH-related HCC patient liver tissue increased significantly compared to simple fatty liver patients and NASH patients^36^. They speculated that ROS production not only promotes inflammation but also promotes hepatocarcinogenesis, and concluded that 8-OHdG is useful for predicting NASH-related HCC development^20^. In the present study, 8-OHdG was significantly elevated in the HFD-60w-*P.g.* (+) group. This implies that increased TNF-α-positive hCLSs in the liver of HFD-*P.g.* (+) group promote NASH-related nodule formation through the oxidative DNA damage.

Integrins are receptors of various extracellular matrices and consist of α and β subunits^37^. *P.g.* binds to integrin α5β1 in oral epithelial cells and leads to integrin signaling activation and cell invasion^23^. Interestingly, *Helicobacter pylori* binds to integrin α5β1 and is involved in stomach cancer development by activating FAK^38^. Integrin β1 signaling has been reported to be involved in HCC development/progression^24,25^. Moreover, drug resistance in HCC is correlated with integrin β1–FAK/AKT signaling^25^. Therefore, we focused on the integrin β1 pathway as another possible mechanism. We found that phosphorylations of FAK, AKT, and ERK were enhanced in *P.g.*-infected cells, in which cell proliferation and migration were upregulated, whereas apoptosis was downregulated. Integrin β1 knockdown significantly reduced the proliferation of *P.g.*-infected cells, probably because integrin β1 signaling was suppressed and only *P.g.*-induced damage to hepatocytes remained. *P.g.*-infection also induces anti-apoptotic effects and activates cell proliferation by activating the AKT signaling pathway in gingival epithelial cells^39^. In particular, AKT plays a central role in various signal networks and is attracting attention as a therapeutic target for HCC^40^. Surprisingly, the total AKT in the NASH liver of the HFD-60w-*P.g.* (+) group was significantly upregulated. It is reported that AKT1 expression is higher in HCC cells than in normal hepatocytes, and AKT1 suppresses the phosphatase and tensin homolog and upregulates Notch1 to cause HCC growth and anti-apoptosis^41^. However, the mechanism by which *P.g.*-infection induces the upregulation of the total AKT in the NASH liver remains unclear. This should be clarified in the future.

This study has potential limitations. Since in this experiment, samples size are fairly small and/or observation period is too short, we could not proof the significant difference in HCC development rate between HFD-*P.g.* (−) and HFD-*P.g.* (+) mice. Future study to clarify the important promoting effects of *P.g.*-odontogenic infection on HCC-development in HFD mice is needed. And pathologic scoring systems are limited by human error.

Moreover, these results were only confirmed by experiments using cell culture and animal model. To clarify the effects of *P.g.*-odontogenic infection on human NASH-related HCC, etiological research such as analysis of tumor characteristics in human NASH-derived HCCs with and without *P.g.* infection, repeated analysis with the addition of a TNF-α blocking agent, and or prospective clinical research is needed in the future.

## Conclusion

**Figure 7:**
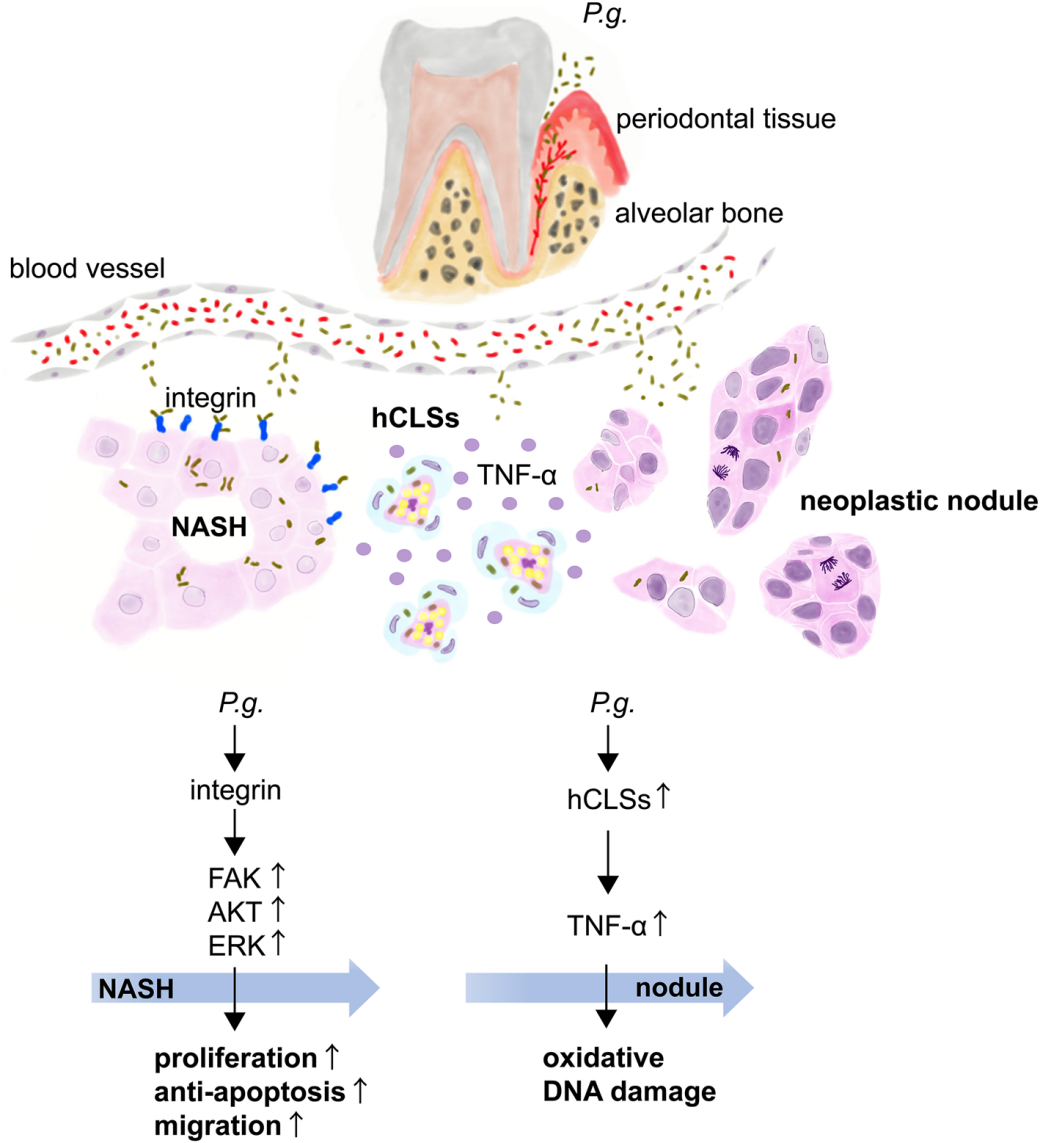
Schematic representation of the mechanisms in which *P.g.*-odontogenic infection promotes NASH-related nodule formation in HFD-induced NASH mouse model. Underlying mechanisms include (1) integrin signaling, i.e., *P.g.* activates integrin signaling including FAK, AKT, and ERK, which promotes proliferation, anti-apoptosis, and migration, and (2) TNF-α produced from hCLSs may also induce activation FAK, AKT and ERK, which are downstream of integrin-signaling, and accelerate proliferation, anti-apoptosis and migration of *P.g.*-infected hepatocytes. TNF-α production leads to oxidative DNA damage and mutagenic 8-OHdG production involved in nodule formation. The two mechanisms finally result in nodule formation. *TNF-α* tumor necrosis factor-α.

Our data suggests that *P.g.*-odontogenic infection might accelerate NASH-related neoplastic nodule formation by activating cell proliferation, anti-apoptosis, and cell migration via the integrin β1-signaling pathway such as FAK, AKT and ERK, and by oxidative DNA damage caused by TNF-α produced from increased Mφ that formed hCLS (Fig. 7).

## Methods

All data generated or analyzed during this study are included in this published article and its supplementary information files.

### Animal experiment

HFD-induced NASH mouse model was developed. To develop fatty liver, the 5-week-old mice (C57BL/6J mice) were fed with HFD (HFD-60; Oriental Yeast Co., Ltd., Tokyo, Japan) for 8 weeks. *P.g.*-odontogenic infection was induced in half of the mice, which were divided into the HFD-*P.g.* (+) group (12 mice) and HFD-*P.g.* (−) group (12 mice). Mice with Chow diet (CD) feeding was also divided into the CD-*P.g.* (−) group (9 mice) and CD-*P.g.* (+) group (9 mice) (Supplementary Fig. 4a). 61-week-old HFD-48w-*P.g.* (−) group (5 mice) and HFD-48w-*P.g.* (+) group (10 mice) were also prepared. For *P.g.*-odontogenic infection, under intraperitoneal anesthesia using pentobarbital sodium (1.62 mg/30 g, Kyoritsu Seiyaku Co., Tokyo, Japan), midazolam (0.06 mg/30 g, B.W. Sandoz K.K., Tokyo, Japan) and diazepam (0.0045 mg/30 g, B.W. Sandoz K.K.), the occlusal surfaces of bilateral upper first molars of the mice were opened with a # 1/2 round bar (J. MORITA CORP., Osaka, Japan). Then, the pulp chamber ceiling and coronal pulp was removed. Then the self-etching primer and a bonding material (Kuraray Noritake Dental Co., Tokyo, Japan) were applied, and light irradiation was done for hardening, and a small cotton ball impregnated with 1 μl of PBS containing 10^8^ *P.g.* strain W83 was put into the cavity and sealed with a photopolymerizable composite resin (Kuraray Noritake Dental Co., Ltd.) (Supplementary Fig. 4b).

Periodontal tissues and livers were evaluated at 60 weeks after *P.g.*-odontogenic infection immunohistochemically and histomorphometrically.

This study was conducted in strict accordance with the recommendations of the Guide for the Care and Use of Laboratory Animals of the Hiroshima University Animal Research Committee and AVMA Guidelines on Euthanasia. This experimental design was approved by the Hiroshima University Animal Experiment Ethics Committee (permit no. # A17-2). This study complied with ARRIVE 2.0 guidelines for preclinical animal study. All mice were housed in a specific pathogen-free facility in 12-h light–dark cycles with access to water and food ad libitum, and health monitoring was conducted daily. All mice were male. Mice were divided into four groups at random. Up to five mice were kept in one cage (1–3 mice: small cage, 4, 5 mice: large cage).

In order to exclude the possibility of the carcinogenic effects on hepatocarcinogenesis induced by chronic inflammation of the injured area.

One CD-60w-*P.g.* (−), one CD-60w-*P.g.* (+), four HFD-60w-*P.g.* (−) and one HFD-60w-*P.g.* (+) mice were excluded because they were injured due to fighting or diffuse ventral decubital ulcer caused by cage in too much obese.

#### Bacterial culture

*P.g.* used in the present study was kindly provided by Dr. Kazuhisa Ouhara (Hiroshima University). *P.g.* was cultured for 4 days at 37 °C under anaerobic conditions using Anero Pack (Mitsubishi Gas Chemical Company).

Optical density (OD value) was measured at a wavelength of 660 nm using a spectrophotometer: SPECTRONIC 200 (Thermo Fisher SCIENTIFIC) to measure the cell concentration of *P.g.* diluted with PBS.

#### Macroscopic analysis of liver nodules

After careful observation of the liver surface, 2-mm sections of each liver lobe were continuously resected. The cut surface was evaluated if there were 1-mm nodules that could be investigated macroscopically. The diameter (mm) of the largest nodule in each mouse was measured, and was categorized as no tumor, < 4 mm or ≥ 4 mm. The area of nodules was calculated and compared using polygon selections of ImageJ software, ver.1.52a (https://imagej.net/ij/docs/index.html). The number of nodules in each mouse was also counted, and was categorized as no tumor, 1, ≥ 2. Nodules were histologically divided into precancerous lesions (score 1) or HCC (score 2). Mice with no nodules were scored as 0 in each nodule profile category. Nodules of HFD-48w-*P.g.* (−) and *P.g.* (+) groups were evaluated with the macroscopic findings of the liver surface because all but one mouse had no nodules.

#### Histopathological analysis

Liver tissues were immersed in PLP fixative for 48 h and immediately embedded in paraffin according to routine methods.

Periodontal tissues were demineralized using 10% EDTA-4Na phosphate buffer solution (Sigma-Aldrich Japan Co., Tokyo, Japan) for 4 weeks after PLP fixation. Then 4.5 μm thickness of paraffin sections were stained with HE. The histopathological diagnosis of the nodules and evaluation of the underlying NASH liver were performed by two pathologists. NAS was calculated based on Kleiner’s criteria (Supplementary Table 1) by scoring steatoses (0–3), lobular inflammation (0–3), and hepatocyte ballooning (0–2) in four pictures at × 200 magnification^16^. Changes in periapical tissue were examined using hematoxylin and eosin-stained specimens.

For histomorphometric analysis, the number of Mac-2-positive Mφ and hepatic crown-like structures (hCLSs) and TNF-α-positive hCLSs were counted using four microscopic photographs (× 200 magnification) of randomly selected areas in the non-neoplastic liver. 8-OHdG was evaluated by total score of nuclear (0–3) and cytoplasmic (0–3) staining using four microscopic photographs (× 200 magnification) of randomly selected areas in the non-neoplastic liver. (0 = none, 1 = focal, 2 = diffuse, 3 = diffuse and strong). IL-6 was evaluated by scoring the degree of staining using four microscopic photographs (× 200 magnification) of randomly selected areas in the non-neoplastic liver. (0 = none, 1 = focal, 2 = diffuse, 3 = diffuse and strong).

#### Immunohistochemical staining

The tissue sections were deparaffinized, immersed in methanol containing 0.3% hydrogen peroxide solution, for removal of endogenous peroxidase for 1 h, and treated with Protein Block (Agilent Technologies Japan, Ltd., Tokyo, Japan) to reduce nonspecific staining. The primary antibodies, i.e. anti-TNF-α (ab 9739, abcam Japan, Tokyo, Japan; overnight), anti-IL-6 (ab208113, abcam Japan, Tokyo, Japan; overnight), anti-8-OHdG (bs-1278R, Bioss Antibodies, Woburn, USA; overnight), anti-*P.g.* (kindly provided by Professor Kazuyuki Ishihara of Tokyo Dental University Microbiology Course; 2 h), anti-Mac-2 (Cat 125402, BioLegend Japan, Tokyo, Japan; 2 h), were applied. After washing with PBS, HRP-labeled polymer conjugated secondary antibody (rabbit: Agilent Technologies Japan, Ltd., rat: NICHIREI BIOSCIENCES INC., Tokyo, Japan) was applied for 1 h. For TNF-α and Mac-2 color development, DAB Peroxidase (HRP) Substrate Kit (Agilent Technologies Japan, Ltd.) was used, and positive products were detected as brown particles. For detection of *P.g.* in green color*,* HistoGreen, Substrate kit for peroxidase (Eurobio Ingen, Les Ulis, France) was employed.

#### Western blotting

Tissue lysate was collected from non-neoplastic liver tissue of each animal. β-Actin, FAK, ERK and AKT protein expression was detected according to the following method. Mice liver tissue were cut into 2 × 2 mm in size and mixed with Tissue Extraction Reagent (Thermo Fisher SCIENTIFIC) including Proteinase inhibitor cocktails (Sigma-Aldrich Japan Co.) and homogenized. After centrifugation of the lysate at 10,000 rpm at 4 °C for 5 min, the supernatant was collected, and the protein concentration was quantitated with a XL-Bradford (integrale, Tokyo, Japan).

In vitro experiment samples were prepared as below. Cells were put on ice and washed with chilled PBS, then lysed them with 1% Triton X-100 (Roche Diagnostics KK, Tokyo, Japan), 10 μg/ml L-1 chlor-3-(4-tosylamido)-4 Phenyl-2butanon, 1 mM DTT, 0.1 mM Na 3 VO 4, 10 μg/ml L-1 chlor-3-(4-tosylamido)-7-amino-heptanon-hydrochloride, 0.1 mM leupeptin and 50 μg/ml phenylmethylsulfonyl fluoride. After centrifugation of the lysate at 13,200 rpm at 4 °C for 20 min, the supernatant was collected, and the protein concentration was quantitated with a Bradford protein assay (Bio-Rad Laboratories, Inc., Virginia, USA).

Thereafter, the sample was dissolved in Laemmli buffer and kept at 100 °C for 3 min and separated by polyacrylamide gel electrophoresis, then transferred to a nitrocellulose membrane (Schleicher & Schuell, Dasse, Germany). Blocking was carried out for 1 h at room temperature using 5% skim milk and the primary antibody was acted and overnight at 4 °C. The following antibodies were used as primary antibodies.

Anti-pFAK-Y397 (#8556, Cell Signaling Technology Japan, K.K., Tokyo, Japan), Anti-FAK (#3285, Cell Signaling Technology Japan, K.K.), Anti-pAKT (#4060, Cell Signaling Technology Japan, K.K), Anti-AKT (#4691, Cell Signaling Technology Japan, K.K.), Anti-phospho-p44/42 MAPK (#4376, Cell Signaling Technology Japan, K.K.), Anti-p44/42 MAPK (#4695, Cell Signaling Technology Japan, K.K.), Anti-Cleaved Caspase-3 (#9664, Cell Signaling Technology Japan, K.K.), Anti-PARP (#9542, Cell Signaling Technology Japan, K.K.), Anti-β-Actin (A2228, Sigma-Aldrich Co).

The secondary antibody was applied at room temperature for 1 h. Anti-mouse IgG, HRP-linked antibody (# 7076, Cell Signaling Technology Japan, KK) or Anti-rabbit IgG, HRP-linked antibody (# 7074, Cell Signaling Technology Japan, KK) was used as a secondary antibody. Bands were visualized by chemiluminescence using ECL western blotting detection system (Western Lightning ECL Pro), (PerkinElmer, INC., Massachusetts, USA). The blots were cut prior to hybridization with antibodies, and cropped images are put in the figures. We put uncropped images of blots with membrane edges visible in Supplemental Information file.

### Laboratory investigations

#### Cell culture

Immortalized human fetal hepatocytes (HC3716-hTERT), kindly provided by Prof. Hidetoshi Tahara (Hiroshima University), were cultured in Hepatocyte Culture Media SingleQuots (Lonza, Walkersville, USA) added with Hepatocyte Basal Medium (Lonza) and supplemented with heat-inactivated 15% fetal bovine serum (Cytiva, Tokyo, Japan).

#### Detection of integrin-β1-signaling pathway molecules by Western blotting

HC3716-hTERT cells were infected with *P.g.* at multiplicity of infection (MOI) of 100. After 15, 30, and 45 min of *P.g.*-infection, protein was collected. Phosphorylations of FAK, AKT, and ERK were detected by Western blotting.

#### Proliferation assay

HC3716-hTERT cells infected with *P.g.* at MOI of 100 were seeded in 24-well plates at a density of 1 × 10^4^ cells/well. The number of cells on days 1 and 5 of infection was counted with the Beckman Coulter Z1 Particle Counter (BECKMAN COULTER Life Sciences, Indianapolis, USA), and the growth rate was determined.

#### Flow cytometry

Doxorubicin (Dox) was used to induce apoptosis. One day after *P.g.*-infection of HC3716-hTERT cells at MOI of 100, Dox was applied for 18 h, and the number of apoptotic cells was analyzed using FACSCalibur (Becton–Dickinson, Tokyo, Japan).

#### Assessment of apoptosis by Western blotting

One day after *P.g.*-infection of HC3716-hTERT cells at MOI of 100, Dox was applied for 18 h, and proteins were collected. Apoptosis markers (cleaved caspase-3 and poly(ADP-ribose)polymerase [PARP]) were examined by Western blotting.

#### Wound healing assay

9 × 10^5^ HC3716-hTERT cells were seeded on a 6-well plate. After 24 h, the cells were infected with *P.g.* at MOI 100 for 2 h. Then, they were washed with phosphate-buffered saline, and the wound area was scratched with a pipette tip. The wound areas were measured at 0 h and 24 h, and the closure areas were calculated using an inverted phase-contract microscope (NIKON INSTECH CO., LTD. Tokyo, Japan; 4× objective). The distances of the 4 closest points in one wound were measured and set to n = 4.

#### RNA interference assay

Integrin β1 siRNA and control siRNA (BONAC CORPORATION, Fukuoka, Japan) transfection into HC3716-hTERT cells was performed using Lipofectamine ™ RNAiMAX Transfection Reagent (ThermoFisher SCIENTIFIC). The sequence of siRNA of Integrin β1 is 5'-cagacatcattccaattgt-3'.

#### Statistical analysis

Results are reported as the mean ± standard deviation (SD). Unpaired t-test, Fisher’s exact test, Mann–Whitney test or Kruskal–Wallis test followed by Dunn’s post hoc test were used to analyze the data with the level of significance set at P < 0.05. Statistical between-group differences were evaluated using analysis of variance with the level of significance set at P < 0.001, P < 0.01 and P < 0.05. All statistical analyses were performed using R (EZR on R commander Version 1.55) or GraphPad Prism 9 for Windows (GraphPad Software, CA, USA).

## Supplementary Information

Supplementary Information 1.

Supplementary Information 2.

## Data Availability

The datasets generated and/or analyzed during the current study are available from the corresponding author on reasonable request.
