# Supplementary material for: Porphyromonas gingivalis-odontogenic infection is the potential risk for progression of nonalcoholic steatohepatitis-related neoplastic nodule formation
Source: Sci Rep. 2023 Jun 8;13:9350. doi: 10.1038/s41598-023-36553-y (PMC10250332; doi:10.1038/s41598-023-36553-y)
Supplement: Supplementary file 1 — Supplementary Information 1. [file 41598_2023_36553_MOESM1_ESM.docx]

***Porphyromonas gingivalis*-odontogenic infection is the potential risk for progression of nonalcoholic steatohepatitis-related neoplastic nodule formation**

Shinnichi Sakamoto, Atsuhiro Nagasaki, Madhu Shrestha, Tomoaki Shintani, Atsushi Watanabe, Hisako Furusho, Kazuaki Chayama, Takashi Takata, Mutsumi Miyauchi

**Supplementary information**


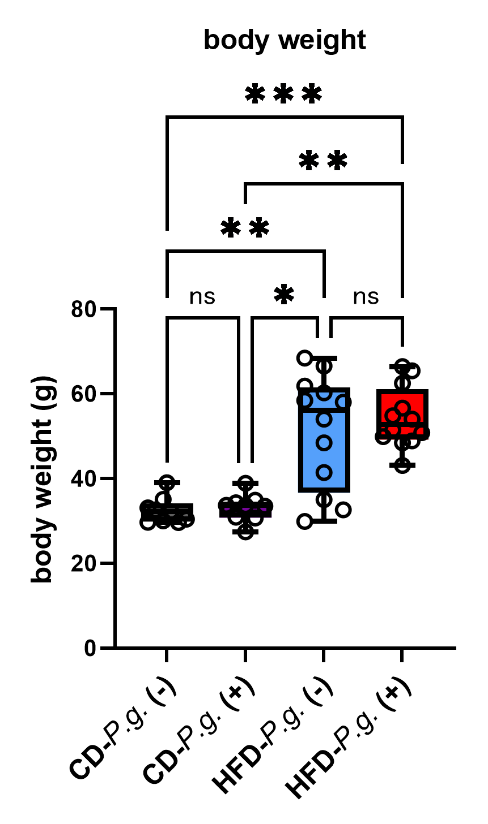

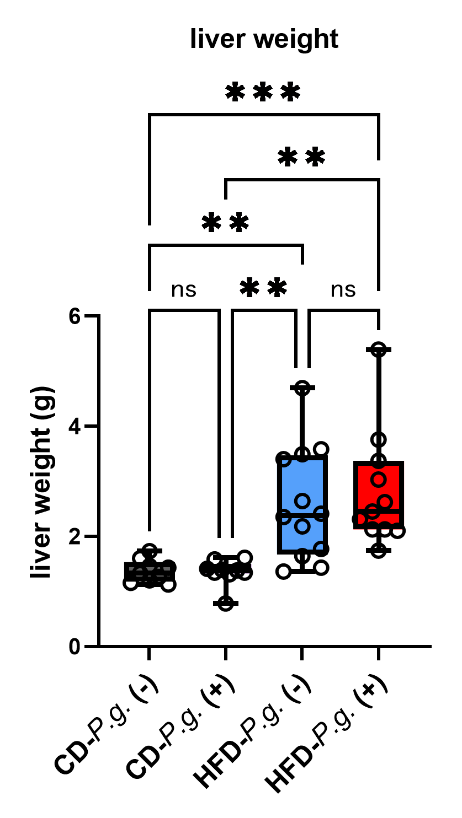


**b**

**a**

Supplementary Fig.1. Body weight and liver weight of mice

Body weight (**a**) and liver weight (**b**) of each group at the time of collection is listed.

body weight. P= 0.0047 CD-60w- *P.g*. (-) vs HFD-60w-*P.g*. (-), P= 0.0006 CD-60w-*P.g*. (-) vs HFD-60w-*P.g*. (+), P= 0.0244 CD-60w-*P.g*. (+) vs HFD-60w-*P.g*. (-), P= 0.0038 CD-60w-*P.g*. (+) vs HFD-60w-*P.g*. (+), P>0.9999 CD-60w-*P.g*. (-) vs CD-60w-*P.g*. (+), P>0.9999 HFD-60w-*P.g*. (-) vs HFD-60w-*P.g*. (+). Liver weight. P= 0.0037 CD-60w-*P.g*. (-) vs HFD-60w-*P.g*. (-), P= 0.0008 CD-60w-*P.g*. (-) vs HFD-60w-*P.g*. (+), P= 0.0057 CD-60w-*P.g*. (+) vs HFD-60w-*P.g*. (-), P= 0.0012 CD-60w-*P.g*. (+) vs HFD-60w-*P.g*. (+), P>0.9999 CD-60w-*P.g*. (-) vs CD-60w-*P.g*. (+), P>0.9999 HFD-60w-*P.g*. (-) vs HFD-60w-*P.g*. (+). (CD-60w-*P.g.* (-) group: n=9, CD-60w-*P.g.* (+) group: n=9, HFD-60w-*P.g.* (-) group: n=12, HFD-60w-*P.g.* (+) group: n=12（body weight), 11 (liver weight)).

**
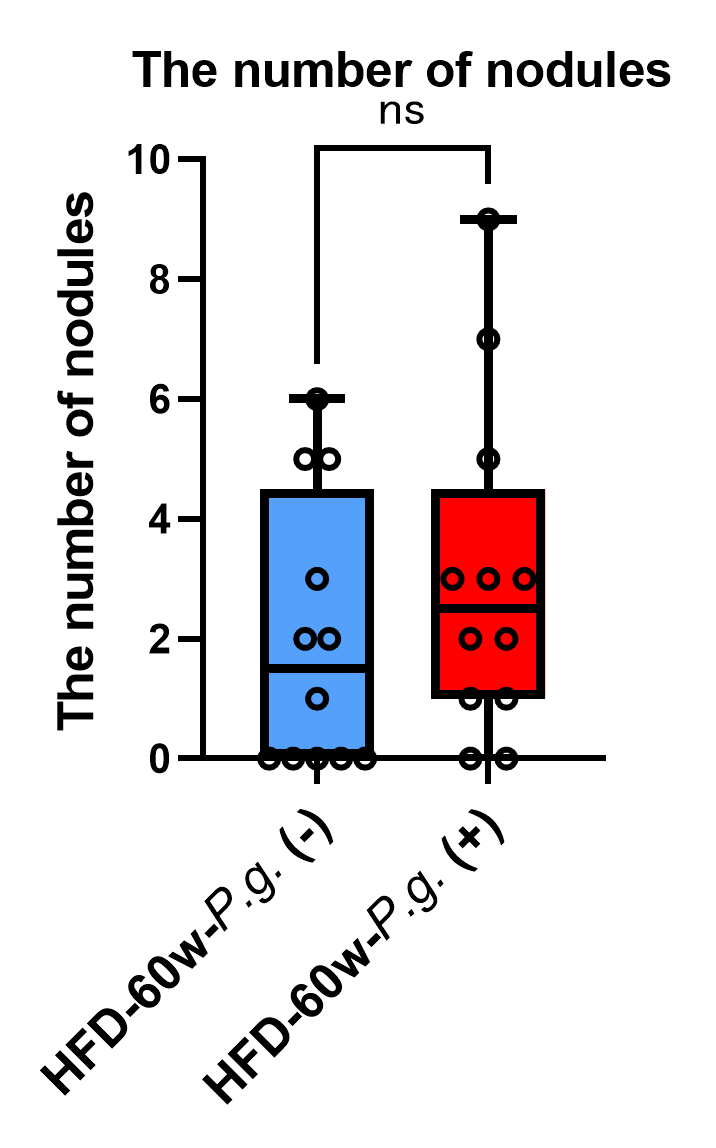
a**

**
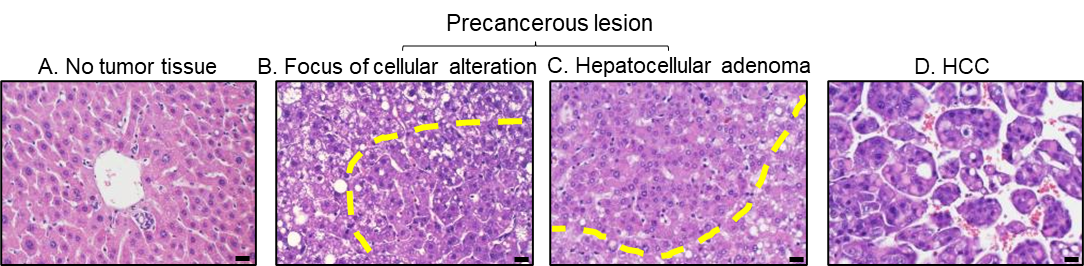
b**

**
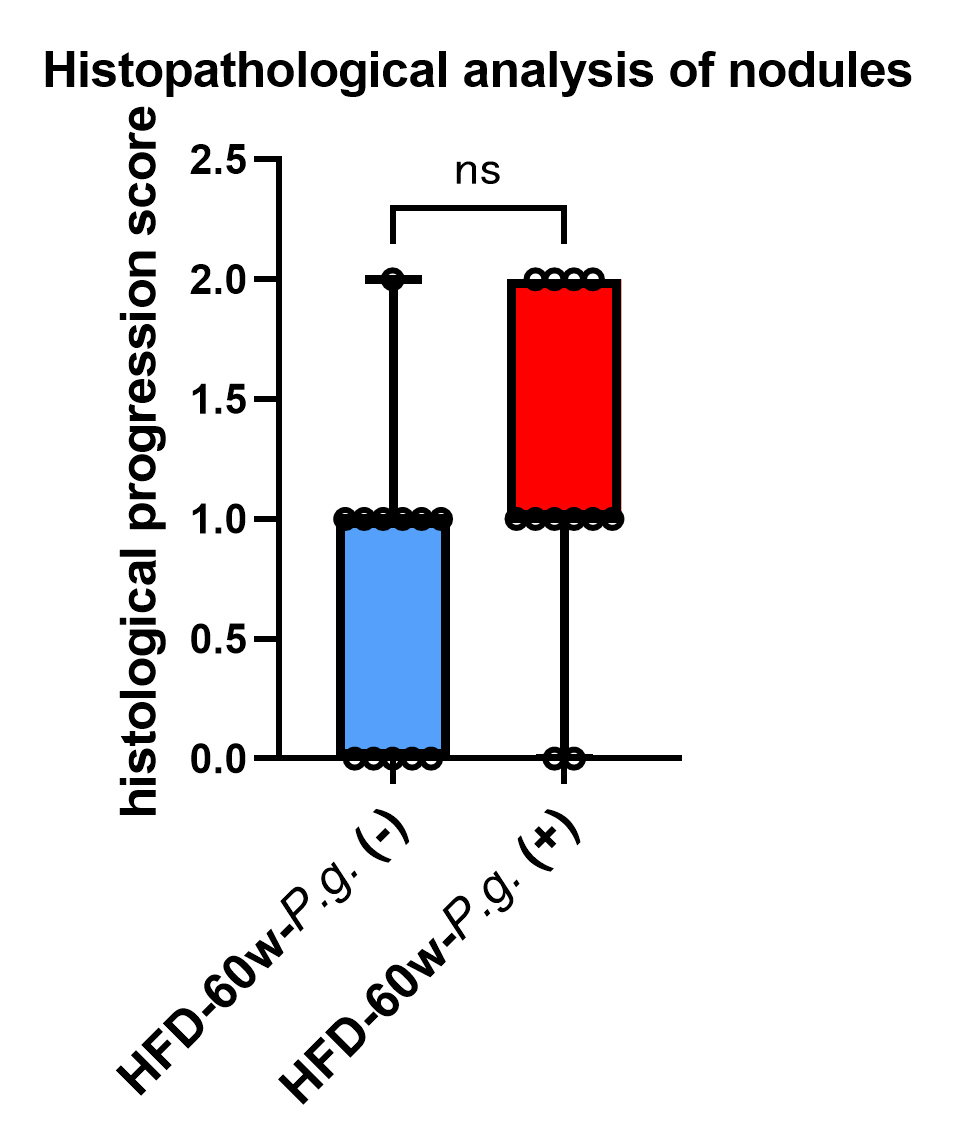
c**

**
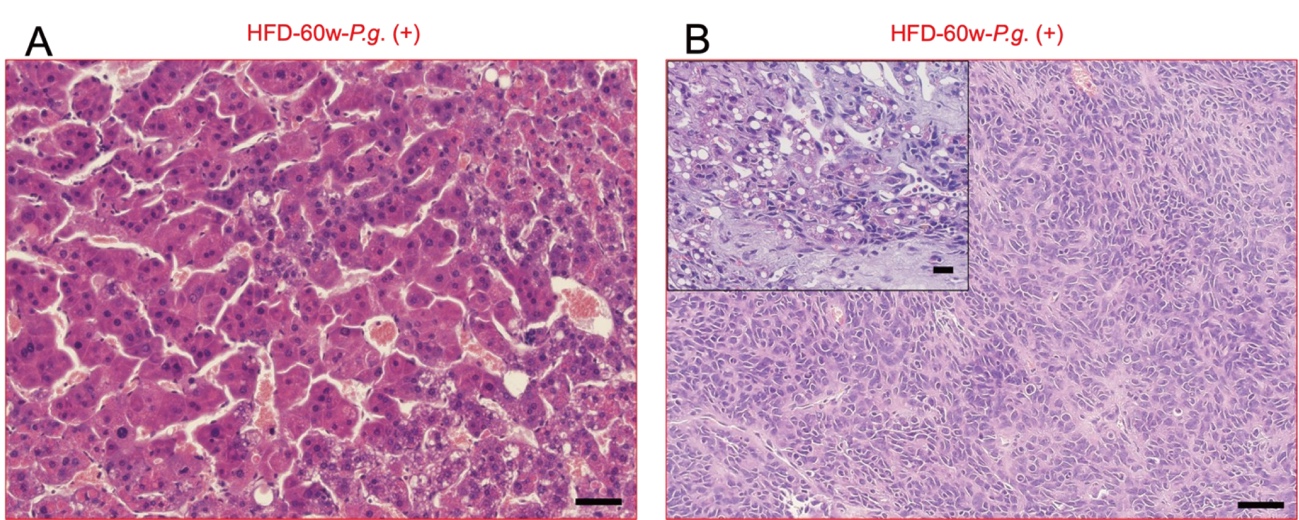
d**

Supplementary Fig.2. Neoplastic nodule profiles in HFD-induced NASH mouse model due to *P.g.*-odontogenic infection

**a**: The number of nodules in the individual mice of the HFD-60w-*P.g.* (-) and (+) groups. P=0.3187; HFD-*P.g.* (-) vs HFD-*P.g.* (+).

**b**: Histopathological process of multistage carcinogenesis in mouse liver.

A: No tumor tissue. B: Focus of cellular alteration (within the dotted line). C: Hepatocellular adcenoma (within the dotted line). D: HCC. (Magnification: ×400. Scale bar=20 µm.)

**c**: Analysis of the histopathological progression. Nodules were histologically divided into precancerous lesions (score 1) or HCC (score 2). Mice with no nodules were scored as 0. P= 0.0956; HFD-*P.g.* (-) vs HFD-*P.g.* (+).

**d**: Histology of HCCs. (A) Trabecular-type HCC is a representative HCC in the HFD-60w-*P.g.* (+) and (-) groups. (B) A hepatoblastoma-like area is developed from trabecular-type HCC in the HFD-60w-*P.g.* (+) group (inset, trabecular HCC component); magnification, ×200; scale bar, 50 µm (insert, ×400; scale bar, 20 µm).

**b**

**a**


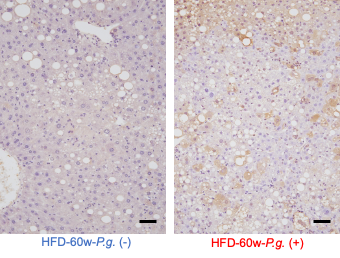


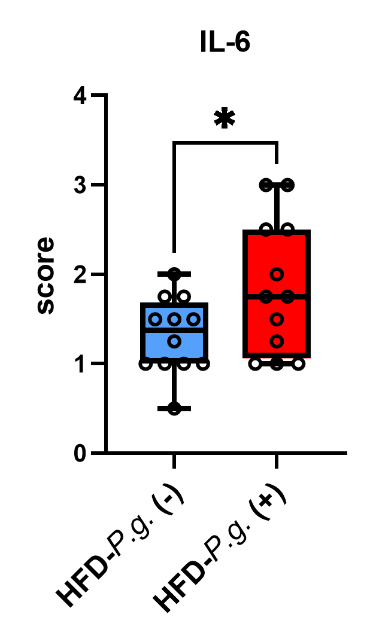


Supplementary Fig.3

**a**: IHC of IL-6 in the non-neoplastic liver tissue of HFD-60w-*P.g*. (-) group and HFD-60w-*P.g*. (+) group ; magnification, ×200; scale bar, 50 µm. **b**: Score of IL-6 (*P<0.05, unpaired t test).


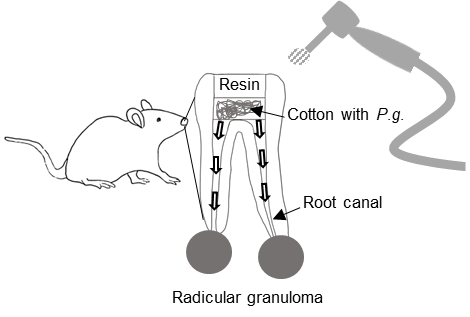

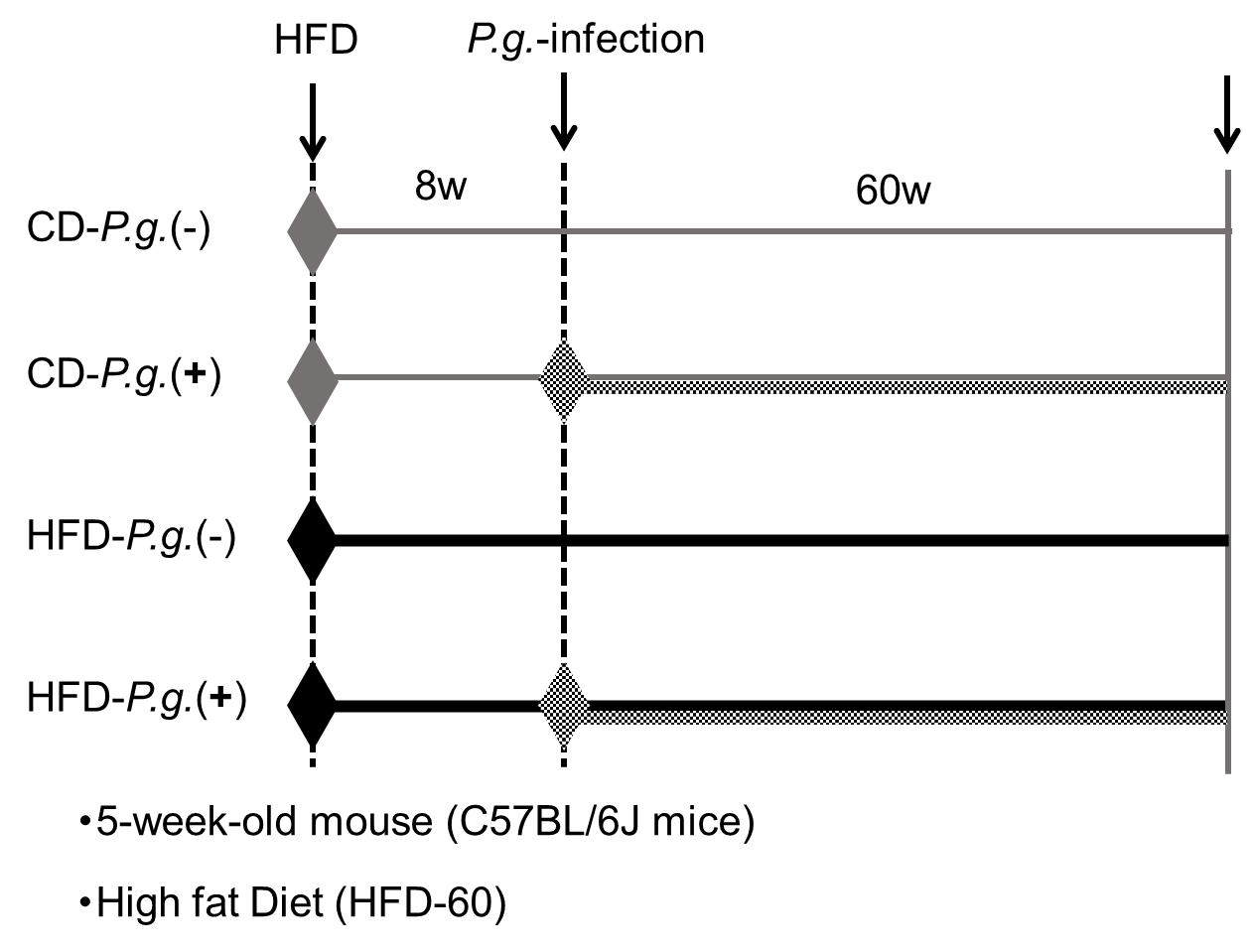


**a**

**b**

Supplementary Fig.4 Animal experimental schedule and schema of the *P.g.*-odontogenic infection method

**a**: Animal experimental schedule for CD and HFD feeding. **b**: the occlusal surface of the upper first molar was opened with a round bar. A small cotton ball with *P.g.* was put into the cavity and sealed with a photopolymerizable composite resin.

**Supplementary Table**

|  | score | definition |
| --- | --- | --- |
| steatosis | 0 | <5% |
|  | 1 | 5-33% |
|  | 2 | >33%-66% |
|  | 3 | >66% |
| hepatocyte ballooning | 0 | none |
|  | 1 | few |
|  | 2 | many |
| inflammation | 0 | No foci |
|  | 1 | <2 foci |
|  | 2 | 2-4 foci |
|  | 3 | >4 foci |

**Supplementary Table.** The NAS scoring system

NAS was calculated based on Kleiner’s criteria. ^1^ According to their report, NAS of ≥5 was regarded as NASH while NAS of 3-4 was borderline NASH and NAS of 0-2 was not NASH. Each score was calculated using four pictures at × 200 magnification. Steatosis: evaluation of parenchymal involvement by steatosis. Hepatocyte ballooning: Few means rare ballooned hepatocytes. Inflammation: Assessment of inflammatory foci.

1 Kleiner, D. E. *et al.* Design and validation of a histological scoring system for nonalcoholic fatty liver disease. *Hepatology* **41**, 1313-1321, doi:10.1002/hep.20701 (2005).
