# Supplementary material for: Porphyromonas gingivalis-odontogenic infection is the potential risk for progression of nonalcoholic steatohepatitis-related neoplastic nodule formation
Source: Sci Rep. 2023 Jun 8;13:9350. doi: 10.1038/s41598-023-36553-y (PMC10250332; doi:10.1038/s41598-023-36553-y)
Supplement: Supplementary file 2 — Supplementary Information 2. [file 41598_2023_36553_MOESM2_ESM.docx]

Fig 6a

pFAK


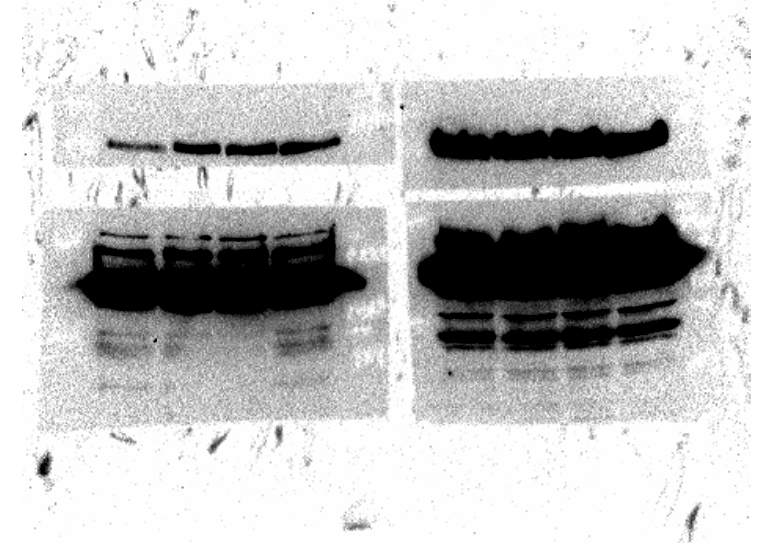


pFAK

136.6 kDa

96.1 kDa

tFAK


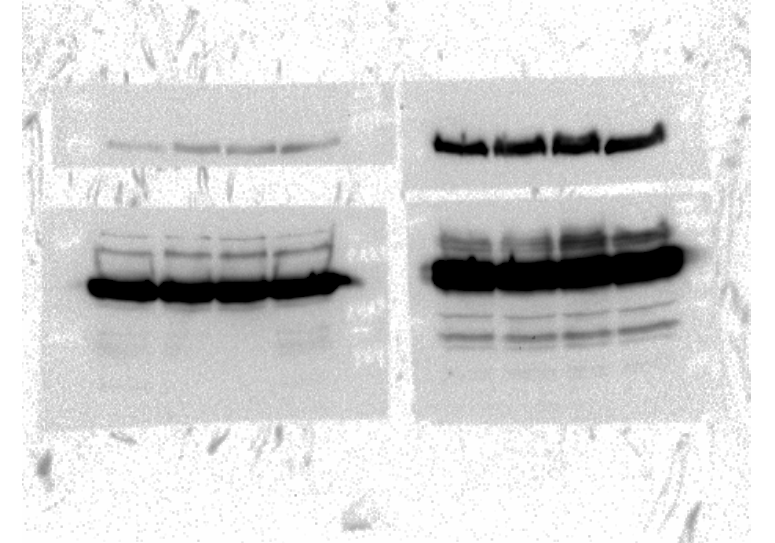


136.6 kDa

tFAK

96.1 kDa

pAKT (Left bands are pAKT. Ohers are another samples. Another contrast image is also attached.)


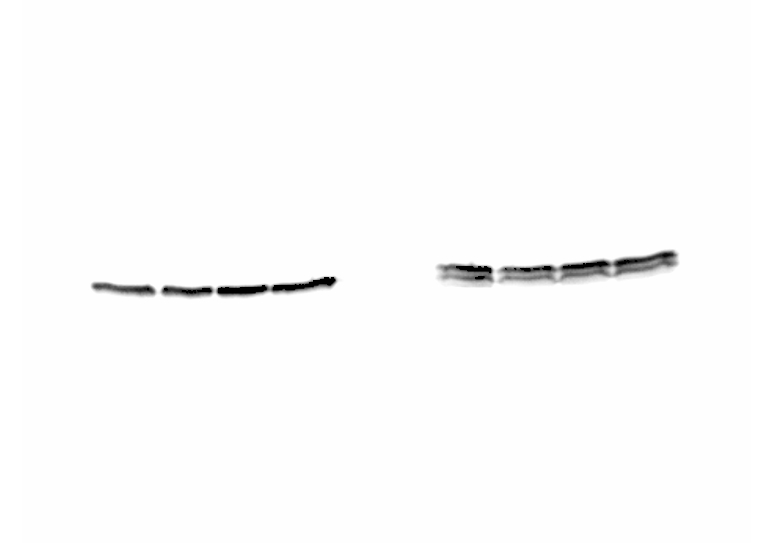


46.2 kDa

72.8 kDa

pAKT


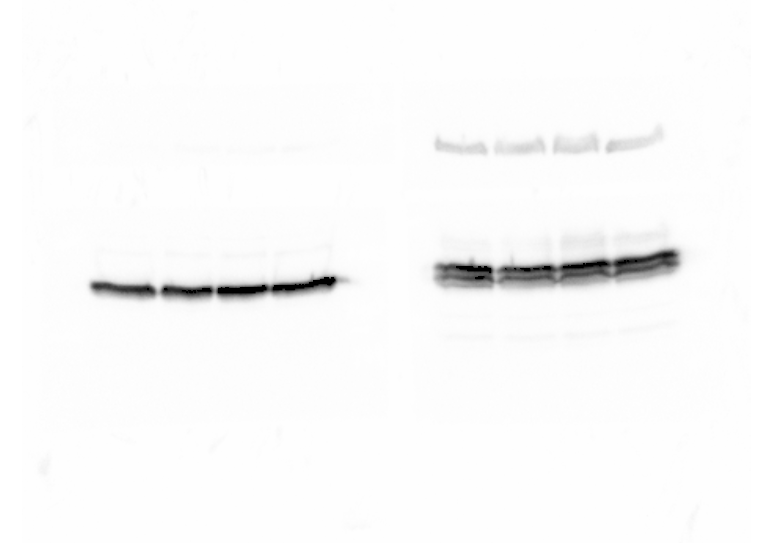


72.8 kDa

46.2 kDa

pAKT


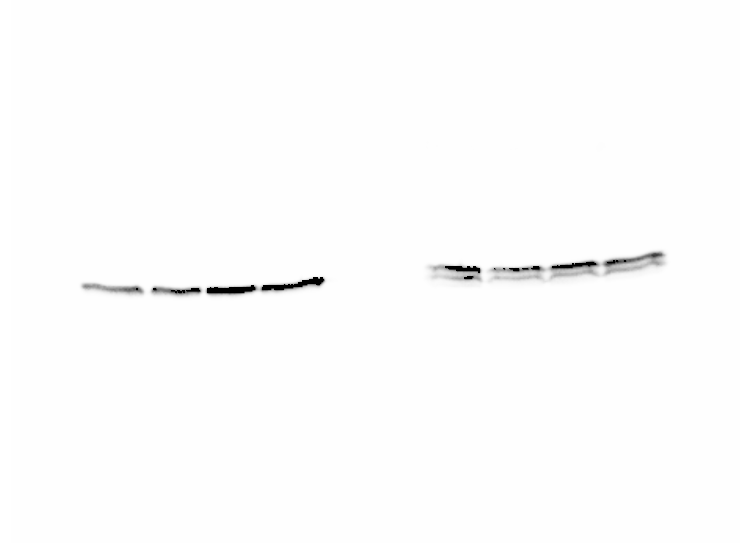


46.2 kDa

72.8 kDa

pAKT


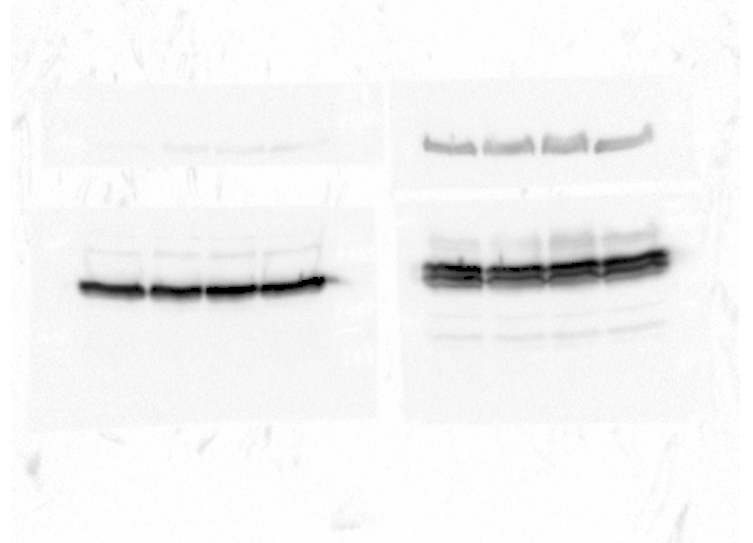


pAKT

46.2 kDa

72.8 kDa


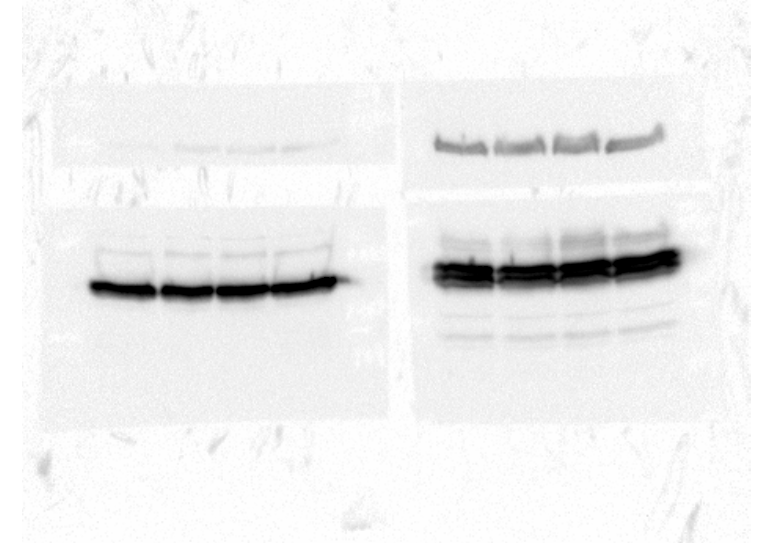
tAKT

tAKT

46.2 kDa

72.8 kDa

pERK
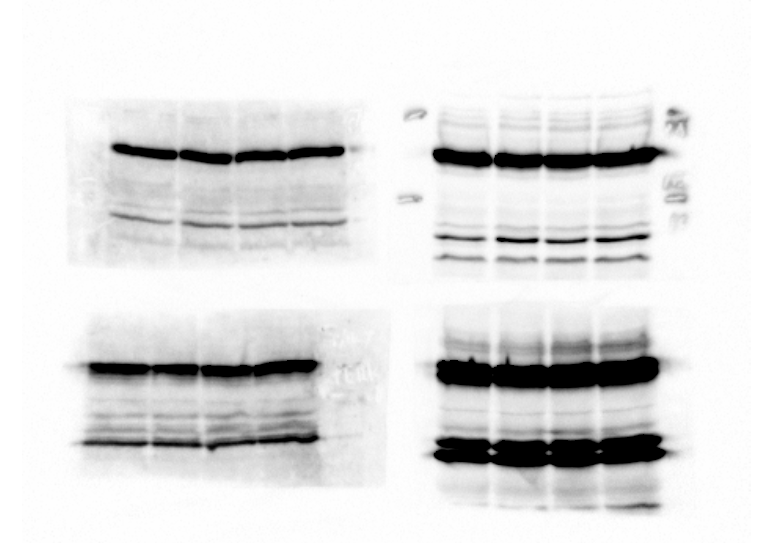


pEAK

46.2 kDa

72.8 kDa


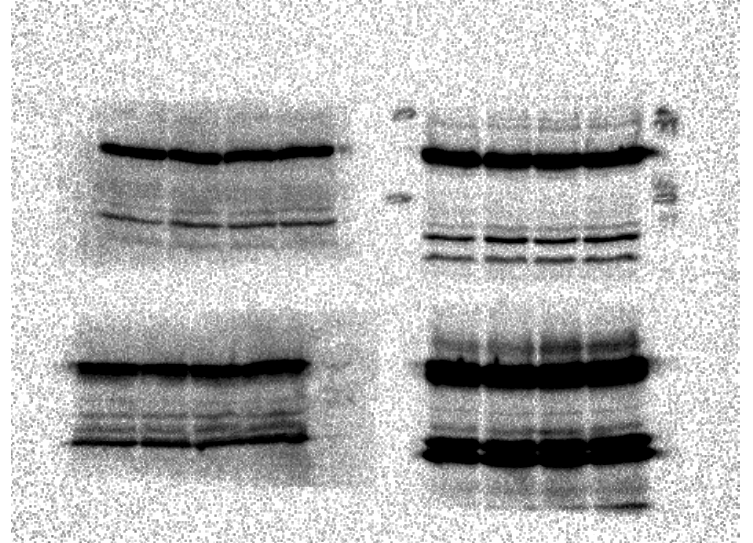


72.8 kDa

46.2 kDa

pEAK

tERK


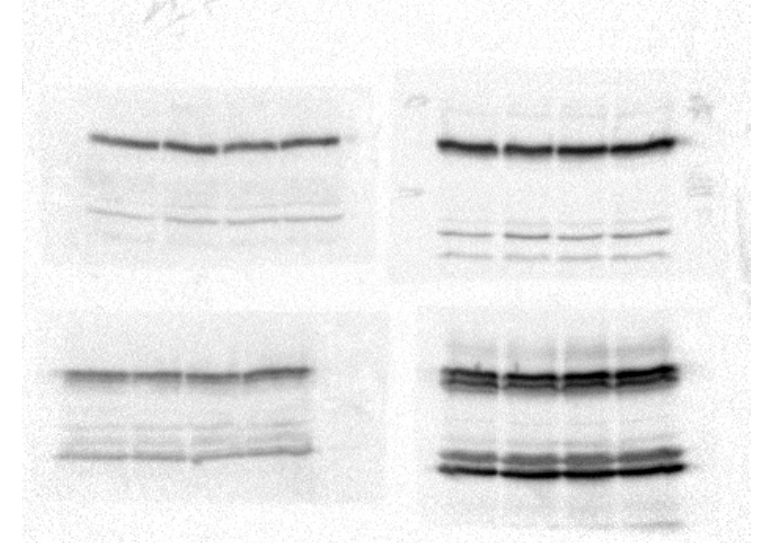


tEAK

46.2 kDa

72.8 kDa

β-actin


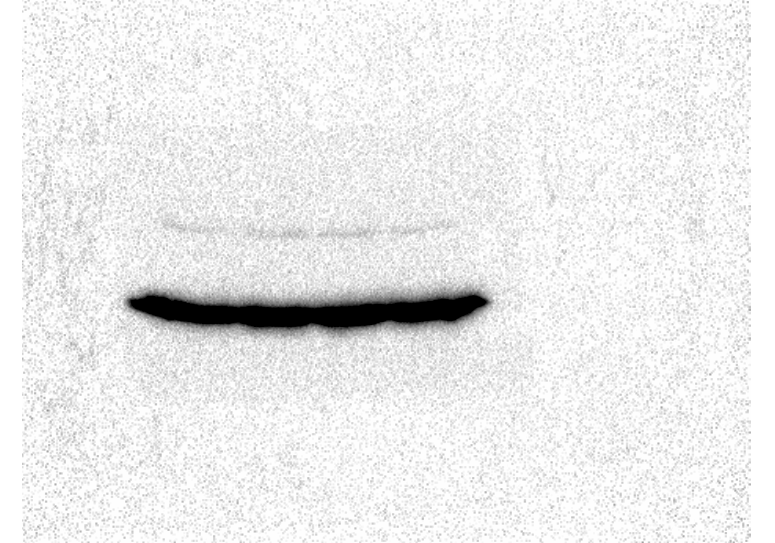


46.2 kDa

72.8 kDa

β-actin


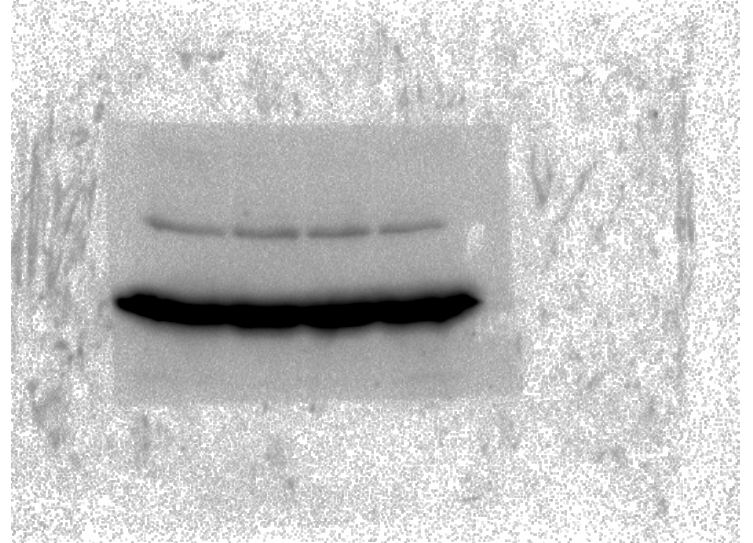


72.8 kDa

46.2 kDa

β-actin

Fig6b

pFAK


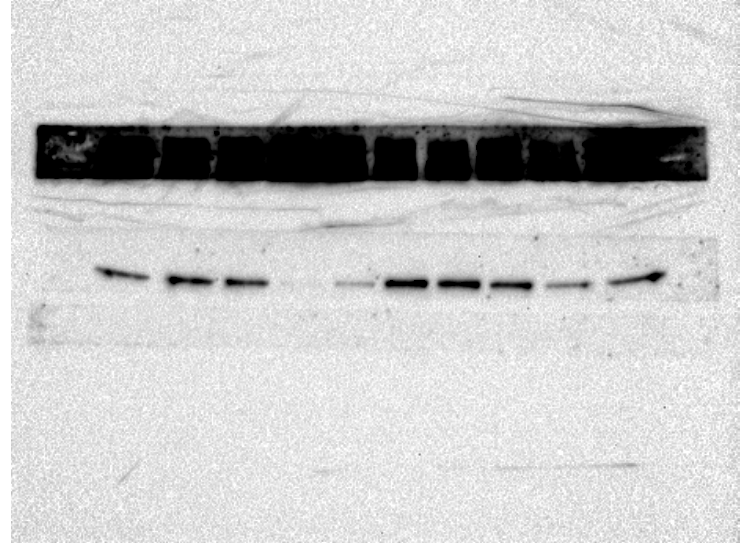


pFAK

136.6 kDa

96.1 kDa

tFAK


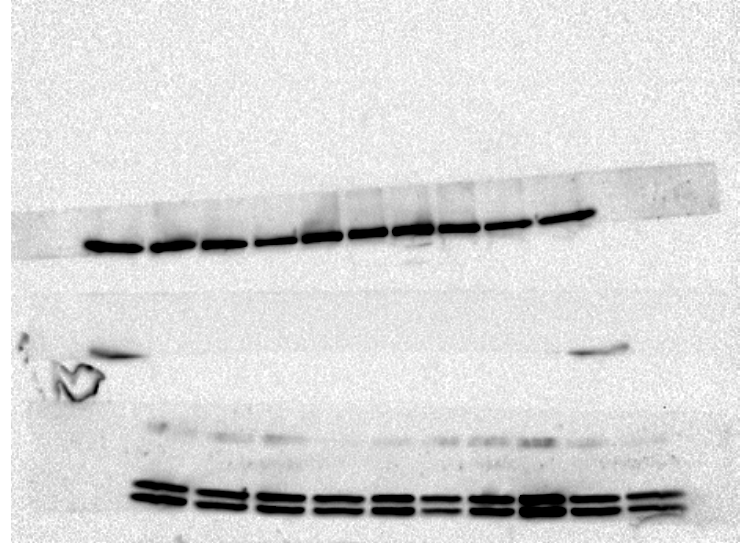


tFAK

136.6 kDa

96.1 kDa

pAKT


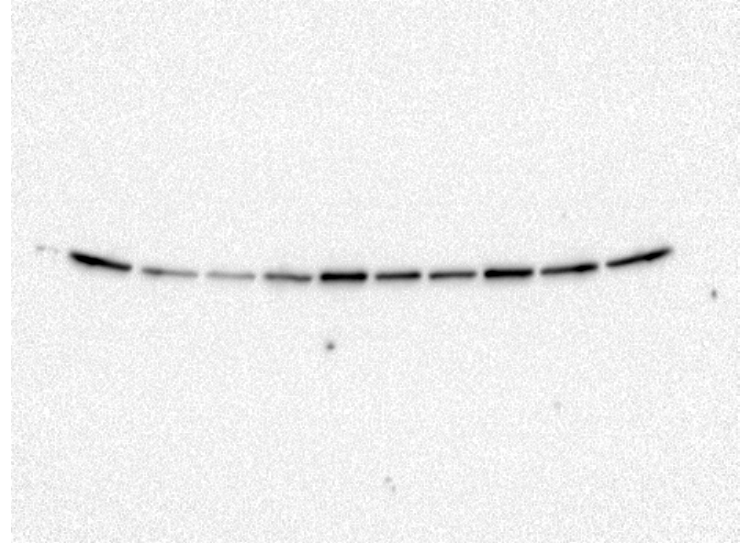


72.8 kDa

pAKT

46.2 kDa


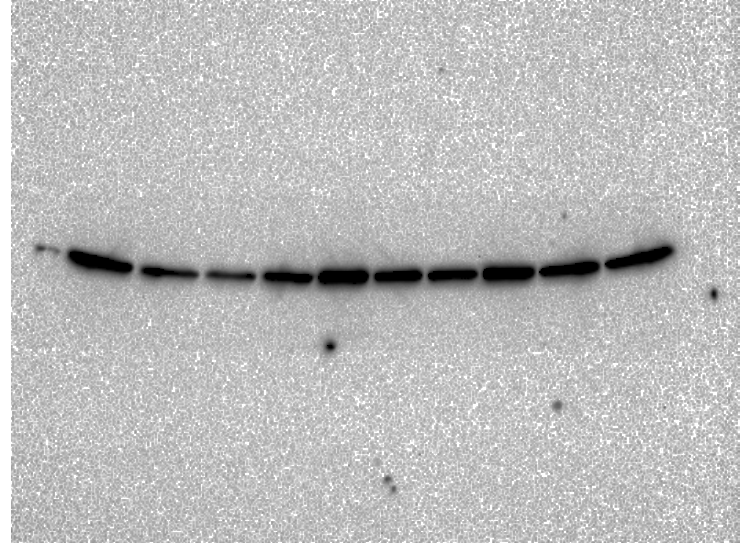


72.8 kDa

pAKT

46.2 kDa

tAKT


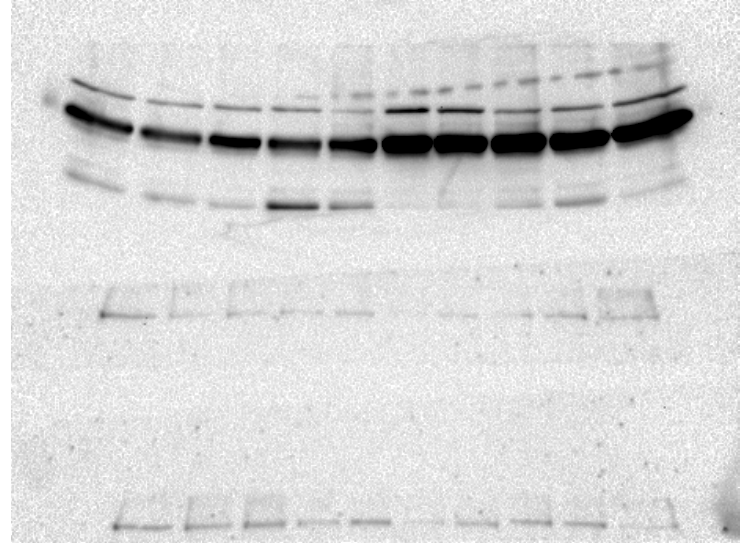


72.8 kDa

tAKT

46.2 kDa


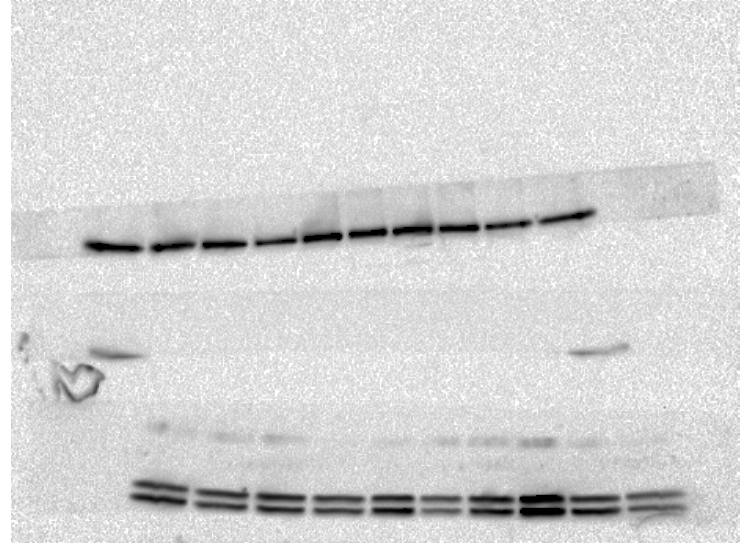
pERK

pEAK

46.2 kDa

72.8 kDa


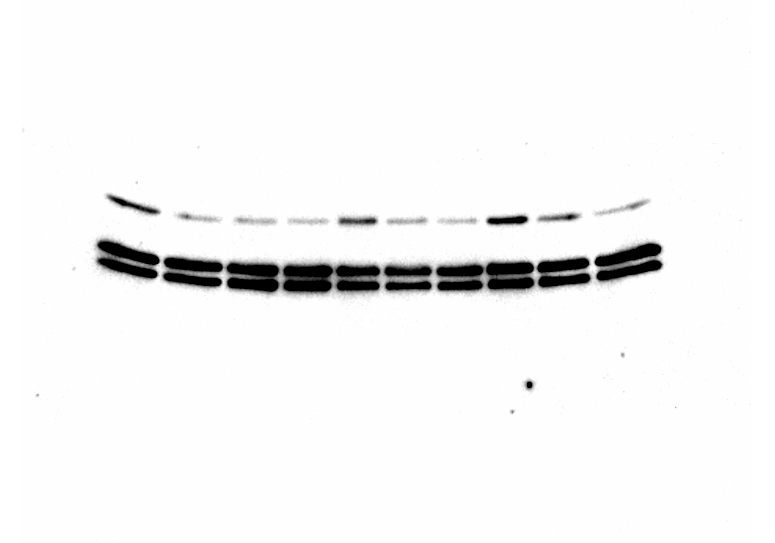
tERK

46.2 kDa

72.8 kDa

tERK

β-actin


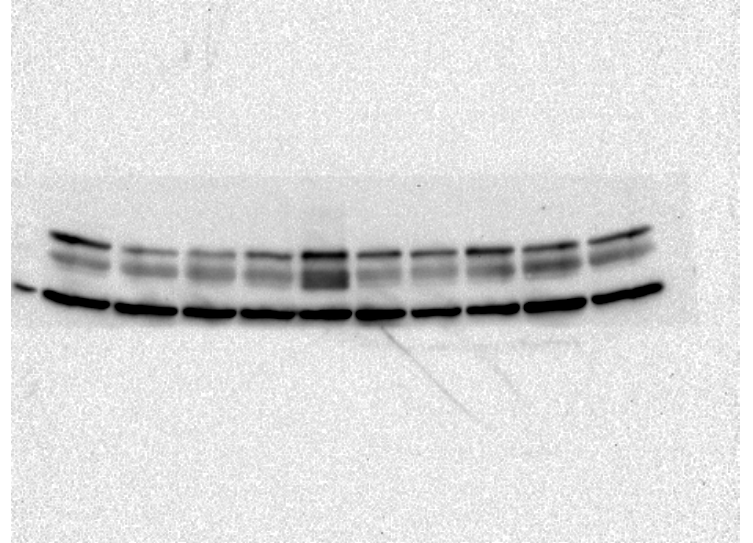


β-actin

72.8 kDa

46.2 kDa

Fig7e

Intβ1

Intβ1

96.1 kDa

136.6 kDa


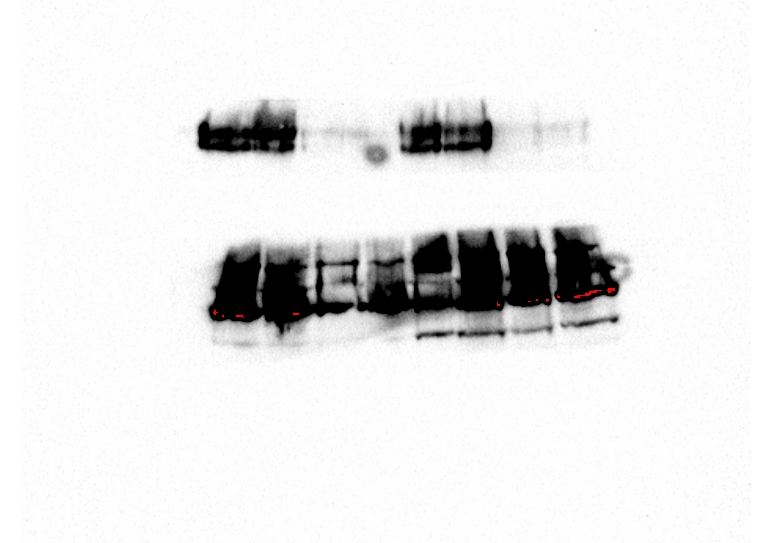


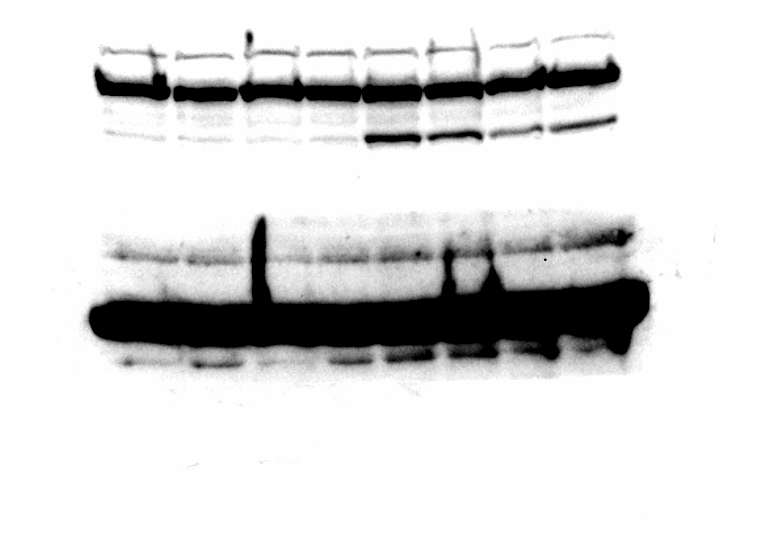


136.6 kDa

96.1 kDa

PARP

cPARP

cCASP3
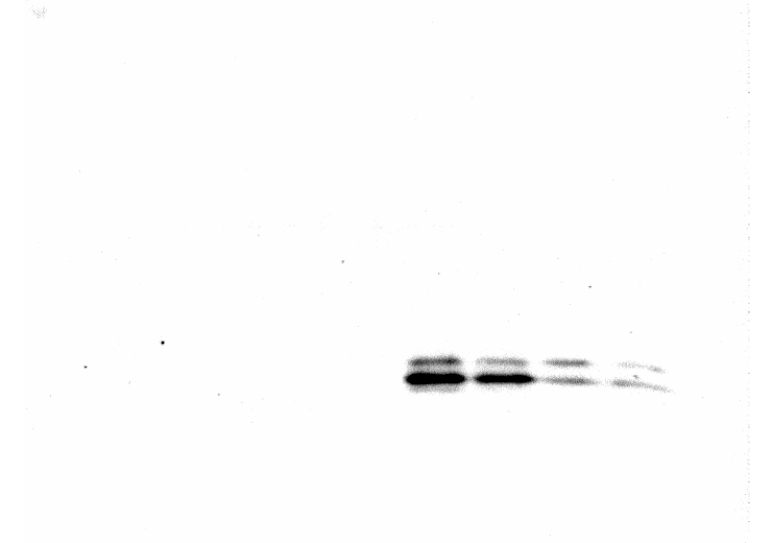


cCASP3

26.3 kDa

17.8 kDa

β-actin


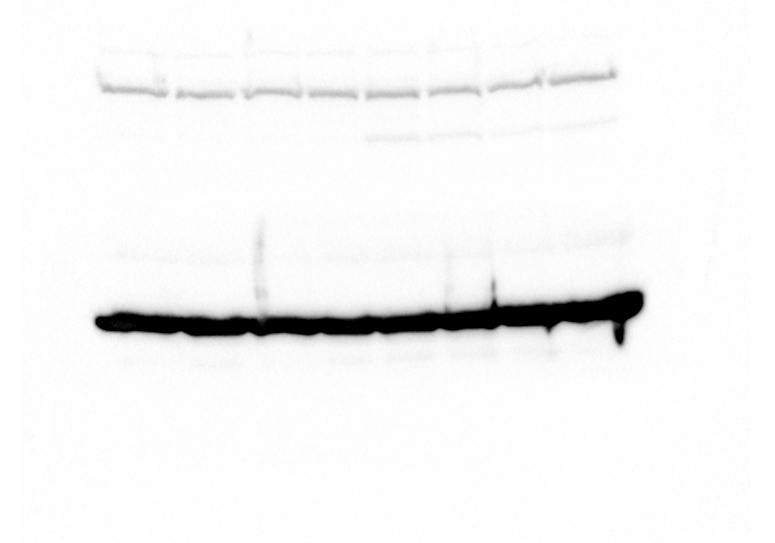


β-actin

46.2 kDa
